# Role of N-linked glycosylation in porcine reproductive and respiratory syndrome virus (PRRSV) infection

**DOI:** 10.1099/jgv.0.001994

**Published:** 2024-05-22

**Authors:** Raymond R.R. Rowland, Alberto Brandariz-Nuñez

**Affiliations:** 1 Department of Pathobiology, College of Veterinary Medicine, University of Illinois at Urbana-Champaign, Champaign, Illinois, USA

**Keywords:** N-glycosylation, PRRSV, viral envelope glycoproteins, viral infectivity

## Abstract

Porcine reproductive and respiratory syndrome (PRRSV) is an enveloped single-stranded positive-sense RNA virus and one of the main pathogens that causes the most significant economical losses in the swine-producing countries. PRRSV is currently divided into two distinct species, PRRSV-1 and PRRSV-2. The PRRSV virion envelope is composed of four glycosylated membrane proteins and three non-glycosylated envelope proteins. Previous work has suggested that PRRSV-linked glycans are critical structural components for virus assembly. In addition, it has been proposed that PRRSV glycans are implicated in the interaction with host cells and critical for virus infection. In contrast, recent findings showed that removal of N-glycans from PRRSV does not influence virus infection of permissive cells. Thus, there are not sufficient evidences to indicate compellingly that N-glycans present in the PRRSV envelope play a direct function in viral infection. To gain insights into the role of N-glycosylation in PRRSV infection, we analysed the specific contribution of the envelope protein-linked N-glycans to infection of permissive cells. For this purpose, we used a novel strategy to modify envelope protein-linked N-glycans that consists of production of monoglycosylated PRRSV and viral glycoproteins with different glycan states. Our results showed that removal or alteration of N-glycans from PRRSV affected virus infection. Specifically, we found that complex N-glycans are required for an efficient infection in cell cultures. Furthermore, we found that presence of high mannose type glycans on PRRSV surface is the minimal requirement for a productive viral infection. Our findings also show that PRRSV-1 and PRRSV-2 have different requirements of N-glycan structure for an optimal infection. In addition, we demonstrated that removal of N-glycans from PRRSV does not affect viral attachment, suggesting that these carbohydrates played a major role in regulating viral entry. In agreement with these findings, by performing immunoprecipitation assays and colocalization experiments, we found that N-glycans present in the viral envelope glycoproteins are not required to bind to the essential viral receptor CD163. Finally, we found that the presence of N-glycans in CD163 is not required for PRRSV infection.

## Introduction

PRRSV is an enveloped single-stranded positive-sense RNA virus [1,2] belonging to the genus *Betaarterivirus*, family *Arteriviridae*, order *Nidovirales* [3]. PRRSV is divided into two distinct species, *Betaarterivirus suid 1* (PRRSV-1) and *Betaarterivirus suid 2* (PRRSV-2) [4]. Both species circulate in US swine herds. Even though both species produce similar clinical signs, PRRSV-1 and PRRSV-2 possess only about 70 % identity at the nucleotide level [5]. PRRSV is now endemic in most swine-producing countries causing approximately $500–600 million per year in losses to US producers alone [6,7]. Clinical signs after infection include reproductive failure during late gestation such as abortions, respiratory distress in young pigs, and poor growth performance [8,9]. PRRSV mainly infects tissue-specific macrophages in the lungs, tonsils, and lymphoid tissues throughout the pig [10]. CD163-positive macrophages, particularly porcine alveolar macrophages (PAMs), are the PRRSV main target cells *in vivo* [10]. Porcine CD163, a cell surface scavenger receptor expressed exclusively in cells of the monocyte-macrophage lineage [11,12], is the essential cellular entry receptor for PRRSV [13].

Previous *in vitro* studies have suggested several host cellular molecules as potential entry mediators and receptors for PRRSV such as CD169, non-muscle myosin heavy chain 9 (MYH9), heparan sulphate, vimentin, DC-SIGN (CD209), CD151, and CD163 [13,15]. However, research involving genetically modified pigs demonstrated that CD163 is the only essential and sufficient receptor for PRRSV infection [13,15]. The exact function of CD163 remains uncertain, but it has been suggested that CD163 cooperates with CD169 in facilitating viral internalization [13,15]. A role of CD163 in mediating viral membrane fusion with the target cells and viral uncoating has also been proposed [13,15].

PRRSV has a genome of approximately 15 kb in length with a 3′-polyadenylated [poly(A)] tail and a 5′-cap. The viral genome is polycistronic and contains at least ten open reading frames (ORFs), which encodes eight structural proteins and 14 nonstructural proteins (nsp1-12) [16]. The genes for the structural virion proteins encode four glycosylated membrane proteins (GP2, GP3, GP4 and GP5), three non-glycosylated envelope proteins (E, ORF5a, and M) and the nucleocapsid protein (N) [17,18]. GP5 and M are the major envelope proteins and exist as a GP5-M heterodimer on the surface of the virion; The heterodimer is held together by a disulphide bond [19,20]. GP2, GP3, and GP4 are minor surface glycoproteins (GPs). The three proteins form a heterotrimer held together by non-covalent interactions [19]. Kappes *et al*. [21] also described the association of the non-structural protein, nsp2, with the virion. Cryo-electron microscopy followed by tomographic reconstruction of purified PRRSV-2 virions revealed the topological features of the PRRSV surface [22]. The surface of the virion is smooth, reflecting the predominance of the short peptide sequences formed by the ectodomains of M and GP5. A small number of protrusions rise above the surface, formed by the large ectodomains of GP2, GP3 and GP4.

GP5 is composed of transmembrane regions and a N-terminal ectodomain that contains several N-glycosylation sites [19]. Depending on the PRRSV strain, the GP5 ectodomain contains 2–5 potential N-linked glycosylation sites [23,25]. N34, N44, and N51 are the main N-glycosylation sites of GP5 in PRRSV-2 [19,24], while PRRSV-1 contains two putative N-glycosylation sites at residues 46 and 53 [19]. It has been reported that lack of N-glycans linked to N44 (PRRSV-2) and N46 (PRRSV-1) in GP5 has a negative impact in the production of infectious progeny virus and significantly decreases viral infectivity [24,26]. GP2, GP3 and GP4 form a heterotrimer complex in the PRRSV envelope that plays an important role in viral infectivity [27,34]. The three GPs contain conserved N-glycosylation sites in both types of PRRSV strains [29]. N-glycosylation sites at N184 of GP2, N42, N50 and N131 of GP3, and multiple locations (N37, 84, 120 and 130) of GP4 are also required for infectious virus production and viral infectivity [27,28]. While it has been suggested that PRRSV-linked glycans are critical structural components in virus assembly by possibly contributing to proper protein folding, it has also been proposed that they are implicated in the interaction with host cells and thus are important for virus infection. Removal of complex-type N-glycans from PRRSV-1 strongly reduced viral infectivity in porcine macrophages [35]. In addition, it has been suggested that sialic acids on GP5 bind sialoadhesin on macrophages, mediating virus binding and internalization [35,37]. In contrast, a more recent report showed that removal of N-glycans from PRRSV-2 does not affect virus infection of permissive cells [38]. Therefore, evidences that N-glycans present in the envelope GPs of PRRSV play a direct role in infection of permissive cells are not compelling. To get more insight into the role of N-glycosylation in PRRSV biology, this work aims to elucidate the specific contribution of the envelope protein-linked N-glycans to PRRSV infection of permissive cells. To this end, we used a novel strategy to modify N-glycosylation of viral envelope proteins that consists of production of monoglycosylated PRRSV and viral GPs with different glycan states. The results showed that removal or alteration of N-glycans from either PRRSV-1 or PRRSV-2 affected virus infection. Specifically, we found that complex N-glycans are required for an efficient infection in cell cultures. Additionally, we found that presence of high mannose type glycans on PRRSV surface is the minimal requirement for a productive viral infection. Our studies also show that that PRRSV-1 and PRRSV-2 have different requirements of N-glycan structure for optimal infection, since removal of high-mannose and hybrid type N-glycans from PRRSV-1 increased the ability of the virus to infect cell cultures. Altogether, these observations suggest that N-glycans are required for an efficient PRRSV infection. Furthermore, we demonstrated that removal of N-glycans from PRRSV has no influence on viral attachment, suggesting that these carbohydrates played a major role in regulating viral entry. Consistent with this finding, by performing immunoprecipitation assays and colocalization experiments, we found that N-glycosylation is not required for the ability of the different viral GPs to bind CD163. Finally, we found that the presence of N-glycans in the CD163 viral receptor is not required for PRRSV infection.

## Methods

### Plasmids

The plasmid pCDNA3.1-CD163-FLAG, which express pig CD163 (GenBank accession number NM_213976.1) fused to a FLAG epitope, was purchased from GenScript. The pCDNA3.1-CD163-FLAG plasmid, a Q5 site-directed mutagenesis kit (New England Biolabs) and specific primers (Table S1, available in the online version of this article) were used according to the manufacturer’s specifications to generate a recombinant plasmid that expressed a CD163 N-glycosylation mutant by substituting all the N-linking sites with Q. The correctness of the construct was confirmed by sequencing and by Western blotting of the expressed protein. The construction of the recombinant plasmids, which express the viral GP2, GP3, GP4 and GP5 from P129 strain [39] fused to a HA epitope, have been described previously [33]. The plasmids expressing the different viral glycoproteins, a Q5 site-directed mutagenesis kit (New England Biolabs) and specific primers (Table S2) were used according to the manufacturer’s specifications to generate recombinant plasmids that expressed viral glycoproteins N-glycosylation mutants by substituting all the N-linking sites with Q. The correctness of the constructs was confirmed by sequencing and by Western blotting of the expressed proteins.

### Viruses and cells

African green monkey kidney cells MARC-145 (RRID:CVCL_4540) and HEK 293 T cells (ATCC CRL-3216 from American Type Culture Collection, Rockville, MD) were cultured at 37 °C in 5 % CO_2_ in Dulbecco’s modified Eagle’s medium (DMEM; Life Technologies) supplemented with 10 % fetal bovine serum (FBS; Gibco), 2 mM l-glutamine (Life Technologies), and antibiotics (100 U ml^−1^ penicillin and 100 mg ml^−1^ streptomycin; Life Technologies). The PRRSV-2 isolate, a P129 strain expressing a green fluorescent protein (GFP) [40], the PRRSV-1 isolate, Lelystad [41], and the type two prototype PRRSV, ATCC VR-2332 [42], were propagated and titrated on MARC-145 cells, as previously described [43]. P129 is a virulent strain of PRRSV isolated in 1995 from an outbreak of highly virulent PRRSV in Southern Indiana, USA [44].

### Antibodies, and reagents

The following reagents and their commercial sources were used: tunicamycin (Sigma), kifunensine (Tocris Bioscience), anti-FLAG-agarose beads (Sigma), and 3XFLAG peptide (Sigma). The monoclonal anti-FLAG M2 antibody was purchased from Sigma. The anti-GAPDH (anti-glyceraldehyde-3-phosphate dehydrogenase; D4C6R), the FLAG tag (D6W5B) rabbit monoclonal antibody, the HA-Tag (C29F4) rabbit monoclonal antibody, and the HA-Tag (6E2) mouse monoclonal antibody were from Cell Signalling. The anti-nucleocapsid monoclonal antibody (SDOW17) was purchased from Rural Technologies Inc. The Alexa 594- and Alexa 488-conjugated antibodies against mouse and rabbit IgG, respectively, were from Thermo Fischer. The anti-CD163 antibody (ab87099) was purchased from Abcam, and the anti-Actin mAb (MA5-11869) was from Thermo Fisher Scientific. The monoclonal anti-GP5 and the monoclonal antibody anti-M protein were a generous gift from Dr Ying Fang (University of Illinois at Urbana-Champaign, USA) [45,46].

### Transfection and immunofluorescence microscopy

Transfections of cell monolayers were performed with the TransIT-LT1 transfection reagent (Mirus) as previously described [32,47]. Transfected cells were incubated at 37 °C for 24 h. For indirect immunofluorescence (IF) microscopy, cell monolayers grown on coverslips were transfected as indicated in the figure legends. At the indicated times, the monolayers were fixed with 4 % paraformaldehyde. Fixed cells were permeabilized with 0.5 % Triton X-100 in phosphate-buffered saline (PBS) and then blocked in PBS containing 2 % bovine serum albumin. After incubation with primary antibodies for 1 h at RT or overnight at 4 °C, the cells were incubated for 1 h with secondary antibodies and DAPI (49,69-diamidino-2-phenylindole). Images were obtained with a Nikon A1R laser scanning confocal microscope. The images were processed with NIS-elements software (Nikon).

### Western blotting

Cellular proteins were extracted with a whole-cell extract buffer (WCE) (50 mM Tris [pH 7.5], 150 mM NaCl, 0.5 % Triton X-100, 10 % glycerol, 1 mM EDTA, protease inhibitor cocktail [Sigma]). Cells lysates were resolved on 4–12% Bis-Tris NuPAGE gels (Invitrogen) and transferred to nitrocellulose membranes using a trans-blot turbo transfer system (Bio-Rad). Protein bands were detected with specific antibodies using SuperSignal West femto maximum sensitivity substrate (Thermo Fisher) and viewed on a FluorChem R system (ProteinSimple).

### Immunoprecipitation assay

Human 293 T cells were co-transfected with plasmids encoding FLAG-tagged CD163, and HA-tagged PRRSV viral glycoproteins variants. At 24 h post-transfection, cells were lysed in 0.5 ml of whole-cell extract buffer. Lysates were centrifuged at 14 000 r.p.m. for 30 min at 4 °C. Post-spin lysates were then precleared using protein A-agarose (Sigma) for 1 h at 4 °C; a small aliquot of each of these lysates was stored as an input sample. Precleared lysates containing the tagged proteins were incubated with anti-FLAG-agarose beads (Sigma) overnight at 4 °C to precipitate the FLAG-tagged proteins. Anti-FLAG-agarose beads were washed three times in WCE buffer, and immune complexes were eluted using 200 µg of FLAG tripeptide per millilitre in WCE buffer without Triton X-100. The eluted samples were separated by SDS-PAGE and analysed by Western blotting using either anti-HA or anti-FLAG antibodies.

### PRRSV titration assay

Monolayers of Marc-145 cells at a confluency of at least 90 % were infected with ten-fold serial dilutions of different PRRSV isolates. At 72 h post-infection, the P129-GFP infected cells were fixed and visualized under a fluorescence microscope. To detect Lelystad and VR2332 virus infections, cells were fixed with 4 % paraformaldehyde. Fixed cells were permeabilized with 0.5 % Triton X-100 in PBS and then stained with PRRSV N-protein-specific mAb (SDOW-17; Rural Technologies Inc.) diluted 1 : 60 in PBS with 1 % FBS (PBS-FBS) (Sigma-Aldrich). After 1 h incubation at 37 °C, the cells were stained with either Alexa Fluor 594-labelled anti-mouse IgG or Alexa Fluor 488-labelled anti-mouse IgG (Thermo Fisher Scientific) diluted 1 : 1000 in PBS-FBS. The plates were incubated for 30 min in the dark at 37 °C, washed with PBS, and viewed under a fluorescence microscope (Nikon ECLIPSE TE2000-S).

### Preparation of growth curves

Monolayers of Marc-145 cells were infected in triplicate with passage three of either P129-GFP or Lelystad isolates at MOI=0.1. After 1 h of incubation at 37 °C, the cells were washed three times with PBS and fresh media was added to the culture. At 24, 48, and 72 h post-infection, cell supernatants were collected to measure the released viral particles by TCID50 analysis. Virus titrations were performed in triplicate on MARC-145 cells. After 3 days, the titration endpoints were determined as the last well showing green fluorescence. The log10 50 % tissue culture infectious dose (TCID50) per millilitre was calculated according to the method of Reed and Muench (1938) [48].

### Quantification and statistical analysis

Densitometric analysis of the blots was performed with ImageJ software (version 2.14.0, National Institutes of Health, USA). All data are presented as mean±standard deviation. The mean and standard deviation values were calculated using GraphPad Prism 7.0 c. Statistical analysis was performed using two-tailed unpaired Student’s t-test. A *P* value of<0.05 was considered to be statistically significant. Number of repeats are specified in the figure legends.

### Treatment with N-glycosylation processing inhibitors and glycosidases

Tunicamycin (TM) and kifunensine (KIF) at a concentration of 1 µg ml^−1^ and 5 µg ml^−1^, respectively, were used to inhibit N-glycosylation in transfected HEK293T cells. Prior to treatment, cell lysates were mixed with glycoprotein denaturing buffer (New England Biolabs) and incubated at 100 °C for 10 min. To remove high-mannose oligosaccharides, cell lysates were treated with 2500 units of endoglycosidase H (Endo H) (New England BioLabs) for 1 h at 37 °C. Then 2500 units of Peptide-N-glycosidase F (PNGase F) (New England BioLabs) was used to remove all N-linked oligosaccharides for 1 h at 37 °C. Digested proteins were analysed by Western blotting with either anti-M2 FLAG monoclonal antibody (Sigma), HA-Tag (6E2) mouse monoclonal antibody (Cell Signalling) or anti-CD163 antibody (Abcam). For glycosidase digestion of viral stocks, PRRSV isolates were incubated with PNGase F or Endo H at 37 °C for 1 h. Mixtures were inoculated into MARC-145 cells or PAMs at an MOI of 1–5 at 37 °C for 1 h. After washing three times with PBS, cells were further incubated for 24–72 h as indicated in the figure legends. Virus without enzyme treatment but containing the buffer-enzyme solution was incubated at 37 °C for 1 h to serve as a temperature stability control. To control for an enzyme effect on cells, Endo H alone was incubated at 37 °C for 1 h. Then, the enzyme was mixed with virus treated at 37 °C for 1 h, and the mixture was added to the cells. To change the N-glycosylation pattern of PRRSV, MARC-145 cells were pre-cultured with 5 or 10 µg ml^−1^ of KIF at 37 °C for 1 h. After removing the medium, cells were infected with PRRSV (MOI=0.1) in the presence of KIF at 37 °C for 1 h. Cells were then washed and further incubated with KIF at the same concentration until virus was harvested. To verify a complete digestion of GP5 by PNGase F, the viral particles were previously mixed with glycoprotein denaturing buffer (New England Biolabs) and incubated at 100 °C for 10 min. Digested GP5 was analysed by Western blotting with a monoclonal anti-GP5 [45].

### Infection of porcine alveolar macrophages (PAMs)

The obtention and infection of macrophages were performed as previously described [49,50]. Lungs were removed from euthanized pigs and lavaged by pouring 100 ml of PBS into the trachea. The tracheas were clamped, and the lungs were gently massaged. The alveolar contents were poured into 50 ml centrifuge tubes and stored on ice. The PAMs were sedimented by centrifugation at 1200 ***g*** for 10 min at 4 °C. The pellets were resuspended and washed once in cold sterile PBS. The cell pellets were resuspended in freezing medium (45 % RPMI 1640, 45 % FBS, 10 % dimethyl sulfoxide [DMSO]) and stored in liquid nitrogen before use. The frozen cells were thawed on ice, counted, and adjusted to 5×10^5^ cells ml^−1^ in medium (RPMI 1640 supplemented with 10 % FBS, PenStrep, and gentamicin). Approximately 10^3^ PAMs per well were added to 96-well plates and incubated overnight at 37 °C in 5 % CO_2_. The cells were gently washed to remove nonadherent cells. Serial 1 : 10 dilutions of PRRSV isolates in medium were added to triplicate wells. At 24 h post-infection, infected PAMs were fixed and stained with PRRSV N-protein-specific MAb (SDOW-17; Rural Technologies Inc.) followed by Alexa 488-conjugated goat anti-rabbit IgG (green) as indicated above. A positive result for infection was recorded as a cell expressing green fluorescence. The infected cells in a 48-well plate were counted, either completely or five to six random fields which corresponds to 500–1000 cells and the total number was calculated from that number [49]. Images of infected PAMs were obtained with a fluorescence microscope (Nikon ECLIPSE TE2000-S).

### Transfection and infection

CD163-FLAG constructs were transfected into PRRSV nonpermissive HEK293T cells using FuGENE HD reagent (Promega) according to the manufacturer’s instructions. Transfected cells were incubated at 37 °C for 48 h. Then, the cells were infected with PRRSV isolates at an MOI of approximately 0.1 or 5. To detect viral infection, cells were fixed with 4 % paraformaldehyde at 3 days post-infection. Paraformaldehyde-fixed cells were permeabilized with 0.5 % Triton X-100 in PBS and then blocked in PBS containing 2 % bovine serum albumin BSA. Then, the cells were co-stained with PRRSV N-protein-specific mAb (SDOW-17; Rural Technologies Inc.) and anti-FLAG tag (D6W5B) rabbit monoclonal antibody in PBS with 2 % BSA. After 1 h incubation at room temperature (RT), the cells were stained with Alexa Fluor 594-labelled anti-mouse IgG and Alexa Fluor 488-labelled anti-rabbit IgG (Thermo Fisher Scientific) in PBS with 2 % BSA. The plates were incubated for 1 h at RT, washed with PBS, and viewed under a fluorescence microscope (Nikon ECLIPSE TE2000-S). The infected cells were detected by the co-localization of green and red fluorescence in the same cell. For each sample, five to six fields were randomly selected in each well to analyse 200–500 single cells for three independent experiments. Colocalization analyses and quantitative analyses of fluorescence channels were performed using ImageJ software (version 2.14.0, National Institutes of Health, USA), as previously described [32,33].

### Virus entry assay

PRRSV viral entry was evaluated as previously described [51]. Monolayers of Marc-145 cells at a confluency of at least 90 % were infected with PRRSV-2 at MOI=1. Since one replication cycle of PRRSV takes 12 h [52], cells were fixed at 9 h post-infection (hpi) and immunostained with PRRSV N-protein-specific mAb (SDOW-17; Rural Technologies Inc.) as indicated above. As secondary antibody Alexa Fluor 594 goat anti-mouse IgG(H+L) (Invitrogen) was used. Cell nuclei were stained with DAPI. Pictures were taken with a Nikon ECLIPSE TE2000-S fluorescence microscope.

### PRRSV quantitative reverse transcriptase (RT)-PCR

Viral RNA was extracted from the supernatant samples using the Ambion’s MagMax 96 RNA Viral Isolation Kit (Applied Biosystems) according to the manufacturer’s instructions. PRRSV RNA was quantified using EZ-PRRSV MPX 4.0 real-time reverse transcription (RT)-PCR target-specific reagents (Tetracore) according to the manufacturer’s instructions as previously described [49]. PCR was performed on a 7500 real-time PCR system (Applied Biosystems) in a 96-well format using the recommended cycling parameters. To quantify viral RNA copies, RNA levels were measured using a standard curve generated from viral RNA isolates included in the kit. The PCR assay results were reported as log_10_ PRRSV RNA copy number per 50 µl reaction volume, which approximates the number of copies per millilitre.

## Results

### Preparation of monoglycosylated virus by using a glycosylation inhibitor and endoglycosidase treatment

**Fig. 1.**
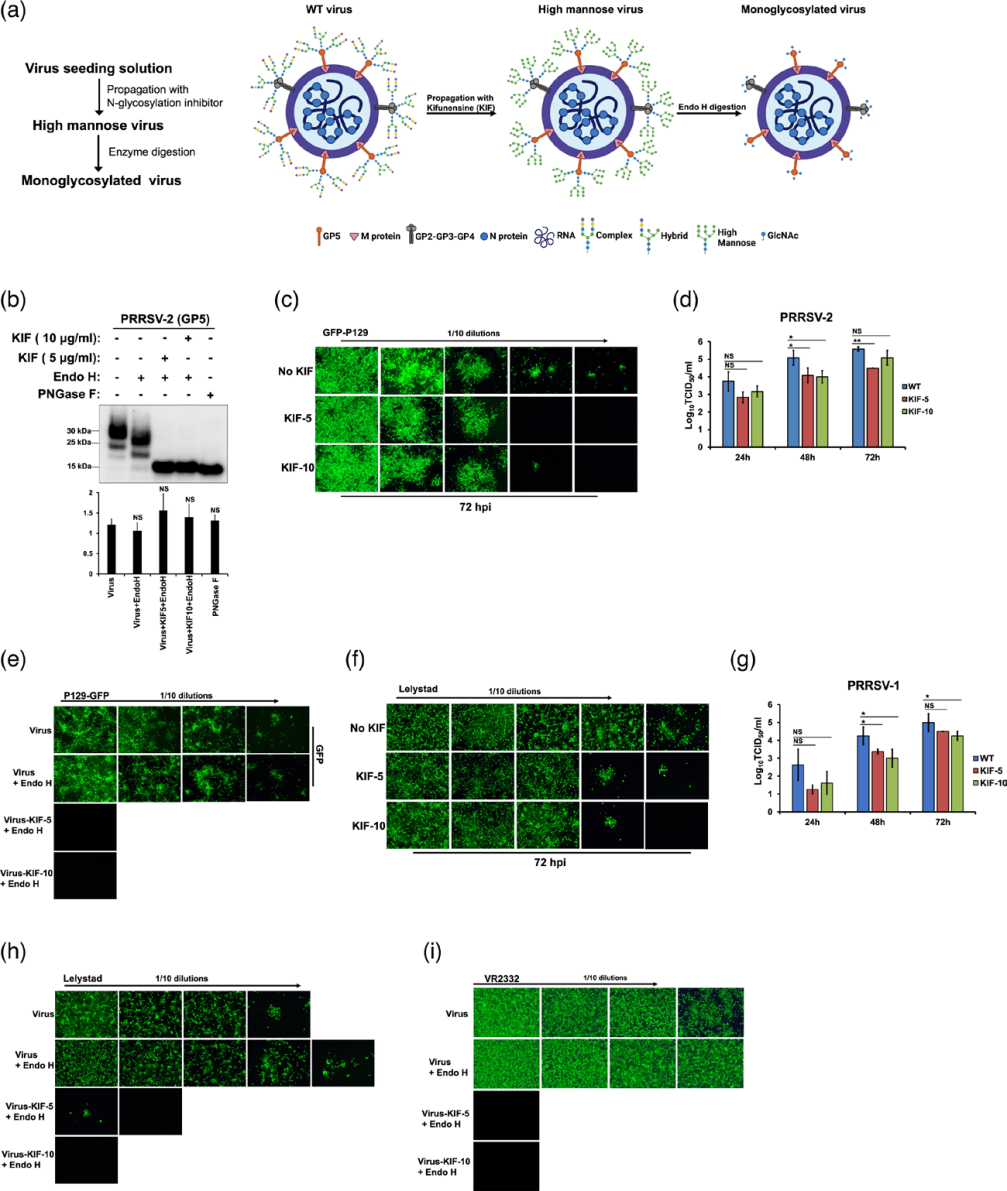
Preparation of monoglycosylated virus. (**a**) Schematic overview of monoglycosylated virus production and viral glycoproteins with different glycan states. Wild-type (WT) virus with the typical complex and hybrid type N-glycans, high mannose virus containing high mannose type N-glycans, and monoglycosylated virus containing N-acetyl glucosamine (GlcNAc) are shown. (**b**) Effect of N-glycosylation inhibition on expression and electrophoretic mobility of viral envelope GP5. Western blot of GP5 expression levels of PRRSV-2 (VR-3223 isolate) propagated in MARC-145 cells with KIF at 5–10 µg ml^−1^ and digested with Endo H or PNGase F. Proteins bands were detected with an anti-GP5 antibody. Densitometry of GP5 bands is shown below the immunoblot. Fold changes are shown relative to lane 1 (virus) and data are mean values± standard deviation (SD) of three independent experiments (NS, not significant). (**c**) MARC-145 cells were infected with different dilutions of P129-GFP virus (starting MOI=1) in absence or presence of KIF at 5–10 µg ml^−1^. At 72 h post-infection (hpi), the cells were fixed and visualized under a fluorescence microscope. Similar results were obtained in three independent experiments and representative data is shown. (**d**) P129-GFP virus (MOI=0.1) was propagated in MARC-145 cells in absence or presence of KIF at 5–10 µg ml^−1^ for 72 h. Cell supernatants were collected at indicated time points to measure the released viral particles by TCID50 analysis. The TCID50 was calculated by titration on MARC-145 cells. Results are means±standard deviation (SD) values from three independent experiments (*, *P*<0.05; **, *P*<0.005; NS, not significant). (**e**) Effect of Endo H digestion on PRRSV-2 infection. P129-GFP propagated in absence or presence of KIF at 5–10 µg ml^−1^ for 3 days was treated with Endo H at 37 °C for 1 h. Different dilutions of virus–enzyme mixtures (starting MOI=1) were inoculated onto MARC-145 cells at 37 °C for 1 h. After infection, cells were washed to remove enzyme and unbound virus, and further incubated for 72 h. Virus without enzyme treatment was incubated at 37 °C for 1 h to serve as a temperature stability control. To control for an enzyme effect on cells, Endo H incubated alone at 37 °C for 1 h was mixed with the temperature-stability control virus and the mixture was added to the cells. Cells were then fixed and visualized under a fluorescence microscope. (**f**) Effect of KIF on PRRSV-1 infection. MARC-145 cells were infected with different dilutions of Lelystad virus (starting MOI=1) in absence or presence of KIF at 5–10 µg ml^−1^. At 72 hpi, the cells were fixed and stained with PRRSV N protein antibody, followed by Alexa 488-goat anti-mouse IgG (green). Similar results were obtained in three separate experiments and representative data is shown. (**g**) Lelystad virus (MOI=0.1) was propagated in MARC-145 cells in absence or presence of KIF at 5–10 µg ml^−1^ for 3 days. Cell supernatants were collected at indicated time points to measure the released viral particles by TCID50 analysis. The TCID50 was calculated as indicated in Fig. 1d. Results are means±standard deviation (SD) values from three independent experiments (*, *P*<0.05; NS, not significant). (**h**) Effect of Endo H digestion on PRRSV-1 infection. Lelystad virus propagated in absence or presence of KIF at 5–10 µg ml^−1^ for 3 days was treated with Endo H at 37 °C for 1 h. Different dilutions of virus–enzyme mixtures (starting MOI=1) were inoculated onto MARC-145 cells at 37 °C for 1 h. After infection, cells were washed to remove enzyme and unbound virus, and further incubated for 72 h. Virus without enzyme treatment was incubated at 37 °C for 1 h to serve as a temperature stability control. To control for an enzyme effect on cells, Endo H incubated alone at 37 °C for 1 h was mixed with the temperature-stability control virus and the mixture was added to the cells. Cells were fixed and stained as indicated above. Similar results were obtained in three separate experiments and representative data is shown. (**i**) Effect of Endo H digestion on VR2332 infection. The same as (**e**) but using a VR2332 isolate. Cells were fixed and stained as indicated above. Similar results were obtained in three separate experiments and representative data is shown.

By performing enzymatic removal of the N-glycosylation sites of PRRSV, it has been proposed that complex-type N-glycans are important for PRRSV infection [35]. In contrast, by realizing similar experiments a more recent study has reported that complex-type N-glycosylation is not required for PRRSV infection on permissive cells [38]. To better understand the role of complex-type N-glycans in PRRSV infection, we used an alternative strategy to modify N-glycosylation of viral envelope proteins that consists of generation of monoglycosylated PRRSV. The schematic diagram in Fig. 1a shows the strategy for monoglycosylated virus production, and the different glycosylation states of viruses generated during the different steps of the process. After the screening of various glycosylation processing inhibitors, kifunensine (KIF) was selected to inhibit the N-glycosylation process to maintain the glycan modification on the viral envelope GPs at high-mannose type [53,54]. KIF is a potent inhibitor of endoplasmic reticulum (ER)-located mannosidase I and complex N-glycosylation, and has been used to block the glycosylation process in cell cultures [47,53, 54]. To test whether KIF is able to arrest the glycosylation state of viral surface GPs at high-mannose type, two different concentrations of KIF not toxic to cells were used [47,55]. To confirm that KIF treatment produce viruses containing high mannose type N-glycans, the viral particles were digested with Endo H, which cleaves only the high-mannose and some hybrid branches of N-glycans, leaving a single N-acetylglucosamine residue [53,54]. Next, the electrophoretic mobility of GP5, the most abundant envelope GP, was analysed by Western blotting using a specific anti-GP5 antibody [45]. Digestion of KIF-propagated viruses with Endo H resulted in a protein band with a molecular weight lower than that of untreated virus (Fig. 1b, lane 3 and 4). Digestion of wild-type (WT) virus with PNGase F, which cleaves all N-glycans, resulted in a GP5 band that migrated with slightly faster electrophoretic mobility than those of KIF-treated viruses digested with Endo H (Fig. 1b, lane 5). This is expected, since digestion with PNGase F produces unglycosylated proteins, while Endo H treatment generates proteins that retain N-acetylglucosamine residues at each of the N-linked glycosylation sites [24]. To confirm a full digestion of GP5 by PNGase F, the viral particles were previously denaturalized by standard methods to ensure a complete digestion by the endoglycosidase (Fig. 1b, lane 5). These results confirm that the KIF treatment generated a virus with high mannose type N-glycans. Moreover, WT virus digested with Endo H resulted in a protein band that migrated with slightly faster electrophoretic mobility than undigested virus but with a bigger size than those of the KIF-treated viruses digested with Endo H, confirming a small presence of high-mannose/hybrid type glycans on GP5 (Fig. 1b, lane 2). These results also confirm that GP5 contains complex N-linked structures since the viral envelope protein is partially resistant to Endo H digestion, which are consistent with previous studies that showed that GP5 contains primarily complex-type N-glycans [24,51, 56]. Altogether, these data confirmed that monoglycosylated virions can be obtained by utilizing the glycosylation inhibitor, KIF, and Endo H digestion.

**Fig. 2.**
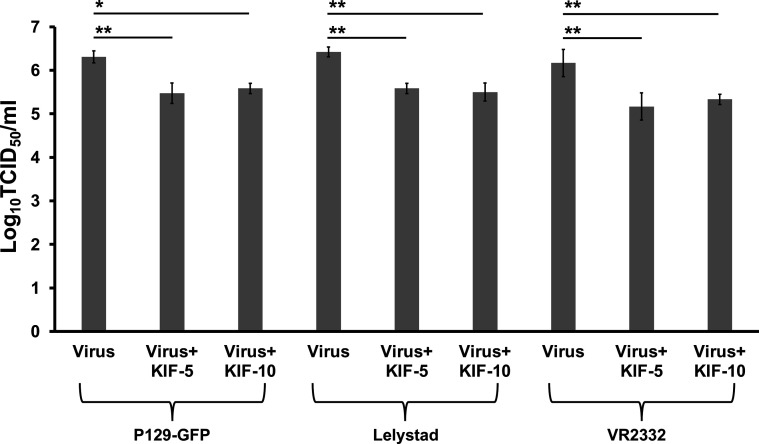
Effect of KIF on PRRSV infection of macrophages. (**a**) PAMs were infected with different dilutions of P129-GFP virus, Lelystad virus or VR2332 virus (starting MOI=1) propagated with KIF at 5–10 µg ml^−1^. At 24 hpi, the cells were fixed and stained as indicated in Fig. 1. P129-GFP infection was visualized directly without antibodies. Virus litres were calculated by titration assay. Results are means±standard deviation (SD) values from three independent experiments (*, *P*<0.05; **, *P*<0.005; NS, not significant).

### Removal or modification of N-glycans from PRRSV-2 affects viral infection

To investigate the role of PRRSV glycans in infection, we examined the effect of inhibition on the formation of hybrid- and complex-type N-glycans on viral infection. For this purpose, we analysed the effect of KIF treatment in PRRSV infection on MARC-145 cells. We used this cell line, since it is highly permissive to viral infection *in vitro*, allowing an efficient replication and growth of PRRSV [57,58]. First, to further validate that KIF treatment induces the formation of high-mannose N-glycans on cultured cells, we evaluated the effect of KIF on the cell surface receptor human ACE2 (hACE2) that mostly contains complex-N-linked structures [47]. As shown in Fig. S1, KIF treatment generated hACE2 that contained exclusively Endo H-sensitive N-glycans, confirming that KIF stimulated the production high-mannose type glycans in cell cultures. Next, we investigated the effect of KIF treatment on viral infection by performing titration endpoint experiments. MARC-145 cells growing in presence or absence of KIF were infected with different dilutions of a highly virulent PRRSV-2 isolate that expresses GFP (P129-GFP) [44]. Viral infection was assessed by fluorescence microscopy, since infection is easily identified by the presence of green fluorescence in infected cells [32,33]. Results showed that untreated cells were infected with the first viral dilution and an endpoint titration of 10^−5^. In contrast, the infection of KIF-treated cells showed titration endpoints of 10^−3^ at KIF concentration of 5 µg ml^−1^ and 10^−4^ at a concentration of 10 µg ml^−1^ (Fig. 1c). Since inhibition of the infection in treated cells was only observed at high viral dilutions, these results suggested that blocking complex N-glycosylation with KIF slightly affects virus infectivity. To analyse whether complex N-glycan are required for an optimal infection, PRRSV was propagated in the presence of KIF. Endo H digestion of virus confirmed that KIF treatment altered glycan types on PRRSV, producing a virus containing high mannose glycan structures (Fig. S2). Next, viral growth curves were performed with the viruses containing high mannose type N-glycans to evaluate the role of PRRSV hybrid- and complex-type N-glycans on infection. As shown in Fig. 1d, WT and modified viruses displayed similar growth curves at 24 h post-infection (h.p.i.). However, the overall yields of the high mannose viruses were nearly one log less than that of WT virus at 48 and 72 hpi. There was no difference in viral growth curves at 72 hpi for virus propagated at KIF concentrations of 10 µg ml^−1^ (Figs1d  3). These findings confirmed that complex-type N-glycans present in PRRSV are required for an optimal infection. Importantly, similar amounts of GP5 were observed in viruses grown in absence or presence of KIF, indicating that KIF treatment did not affect the synthesis of the viral protein (Figs 1b and S2). To further verify that the use of KIF did not influence virus particle formation, the amount of progeny virus in culture supernatant was measured by quantitative real-time PCR as previously described [38,50]. As shown in Fig. S4, there was no difference in the production of virus particles following incubation with KIF compared to virus particles produced without KIF treatment. Next, we analysed whether removal of high mannose glycans from the virus propagated with KIF affects viral infectivity. To this end, KIF-treated viruses were digested with Endo H and titration endpoint experiments were performed as described above. As shown in Fig. 1e, no infection was observed after removing high mannose glycans from KIF-propagated viruses, suggesting that the resulting monoglycosylated virions lost the ability to infect cell cultures. In contrast, treatment of PRRSV with Endo H, which cleaves only high-mannose and hybrid type N-glycans did not affect virus infectivity, suggesting that those kind of glycans are not required for PRRSV infection. Similar results were obtained when infected cells were detected by staining them with an anti-PRRSV N protein antibody (Fig. S5). Altogether, these results suggested that the presence of high mannose type glycans on PRRSV-2 surface is the minimal requirement for viral infection.

### Removal or modification of N-glycans from PRRSV-1 affects viral infection

Since the genotypes of PRRSV-1 and PRRSV-2 share only about 70% at the nucleotide level [5], and GP5 is the most variable envelope protein with only ~50% amino acid homology between PRRSV-1 and PRRSV-2 strains [59,60], we performed the same experiments described above using a PRRSV-1 isolate (Leystad) to analyse the role of N-glycosylation on viral infection. Like PRRSV-2, the results showed that blocking complex N-glycosylation with KIF slightly affects virus infectivity (Fig. 1f). Similarly, our data also showed that complex-type N-glycans present in PRRSV-1 are required for an optimal infection (Fig. S6). In addition, viral growth curves performed with PRRSV-1 containing high mannose type N-glycans showed that WT and modified viruses displayed similar growth curves at 24 hpi. However, the overall yields of the high mannose viruses were nearly one log less than that of WT virus at 48 and 72 hpi. There was no difference in viral growth curves at 72 hpi for virus propagated at KIF concentrations of 5 µg ml^−1^ (Figs 1g and S6). Furthermore, no infection (or very few scattered infected cells at an endpoint titration of 10^−1^ at KIF concentration of 5 µg ml^−1^) was detected after removing high mannose glycans from KIF-propagated PRRSV-1, indicating that the resulting monoglycosylated virions lost infectivity (Fig. 1h). Interestingly, removing high-mannose and hybrid type N-glycans from PRRSV-1 by Endo H digestion increased the ability of PRRSV-1 to infect cell cultures by one log (Fig. 1h), suggesting that PRRSV-1 and PRRSV-2 have different requirements of N-glycan structure for optimal infection. Overall, these results confirmed that the presence of high mannose type glycans on PRRSV-1 surface is the minimal requirement for viral infection.

### Removal or alteration of N-glycans from PRRSV-2 isolate VR2332 affects viral infection

It has been previously shown that enzymatic removal of N-glycosylation sites of the isolate VR-2332 (PRRSV-2) did not reduce viral infection [38]. Therefore, we repeated the same experiments described above using the PRRSV-2 isolate VR2332 to evaluate the role of N-glycosylation on viral infection. Like the P-129 isolate, the results showed that blocking complex N-glycosylation with KIF slightly affects virus infectivity (Fig. S7), and that complex-type N-glycans present in VR2332 are required for an optimal infection (Fig. S8). Similar to the other PRRSV-2 isolate (P129) there was no difference in viral infection at 72 hpi for virus propagated at KIF concentrations of 10 µg ml^−1^ (Fig. S8). Furthermore, no infection was detected after removing high mannose glycans from KIF-propagated VR2332 by Endo H digestion (Fig. 1b), indicating that the resulting monoglycosylated virions lost infectivity (Fig. 1i). Overall, these results confirmed that the presence of high mannose type glycans on PRRSV-2 isolate VR2332 surface is the minimal requirement for viral infection, which is in agreement with our previous results obtained for the PRRSV-2 isolate, P129.

### N-glycosylation inhibition of CD163 receptor does not affect PRRSV infection

Our data showed that pharmacological inhibition of complex N-glycosylation by KIF treatment in cell cultures affected PRRSV infectivity (Figs 1c, f and S7). These results did not rule out the possibility that reduction in viral infection could be due to blocking N-glycosylation of the essential PRRSV receptor CD163 [61,64]. To test this possibility, we analysed the impact of CD163 N-glycosylation on viral infection. Porcine CD163 is a type I transmembrane protein that belongs to the class B of the scavenger receptor cysteine-rich (SRCR) superfamily (SRCR-SF) [13]. The receptor contains seven predicted N-linked glycosylation sites, at amino acid residues 105, 139, 319, 693, 766, 936 and 1106 (Fig. 2a). To understand the role of CD163 N-glycosylation in viral infection, we generated an N-glycosylation-deficient mutant fused to FLAG, in which all the N present in the consensus N-glycosylation sites were replaced by Q (for clarity, this new mutant is herein referred to as CD163-FLAG*). To confirm that the CD163 variant is a N-glycosylation null mutant, we analysed the CD163 expression and electrophoretic mobility after tunicamycin (TM) treatment in transfected cells. TM inhibits the first step of N-glycan biosynthesis, which results in the complete absence of glycan residues [65]. Additionally, digestion of CD163 with PNGase F, which cleaves all N-glycans, was performed. As expected, expression of CD163* resulted in detection of a band with a molecular weight similar to that of TM-treated lysates or samples digested with PNGase F (Fig. 2b, middle panels). Interestingly, TM treatment showed reduced levels of CD163 (Fig. 2b, middle panels). The effect of TM was mimicked by CD163* construct, which lacked glycans (Fig. 2b, left panels). Together, these data suggest that core N-glycosylation is important for CD163 biosynthesis. KIF treatment of transfected cells did not have an impact on the electrophoretic mobility of CD163 and generated bands with a similar molecular weight (Fig. 2b, right panels). Digestion of either KIF-treated or untreated cell lysates with Endo H, which cleaves only the high-mannose and some hybrid branches of N-glycans, resulted in bands at a molecular weight lower than that of untreated controls (Fig. 2b, right panels). These last results confirmed the presence of high mannose/hybrid type glycans on the porcine CD163 receptor. Taken together, these results suggest that CD163 expressed in transfected cells mainly contains some hybrid and high-mannose N-linked structures. In contrast, when the same enzymatic deglycosylation experiments were performed with CD163 from PAMs, we found that treatment with Endo H resulted in two species for CD163: a larger Endo H-resistant form and a smaller Endo H-sensitive form (Fig. 2c). This implies that CD163 contained mostly Endo H-resistant complex oligosaccharides. These results also suggested that endogenous CD163 carries both high mannose and complex type N-glycans. Next, to analyse the role of CD163 N-glycosylation in PRRSV infection, we transiently expressed the N-glycosylation-deficient mutant fused to a FLAG (CD163-FLAG*) in non-permissive cells (Fig. 2e), and subsequently the transfected cells were infected with either PRRSV-1 or PRRSV-2 at different MOIs. As shown in Fig. 2d, removal of N-glycosylation sites did not affect the capacity of CD163 to allow both PRRSV-1 and PRRSV-2 infections, since viral infection was detected in cells expressing the mutant CD163. Altogether, these findings revealed that N-glycosylation of CD163 is not required for PRRSV infection.

**Fig. 3.**
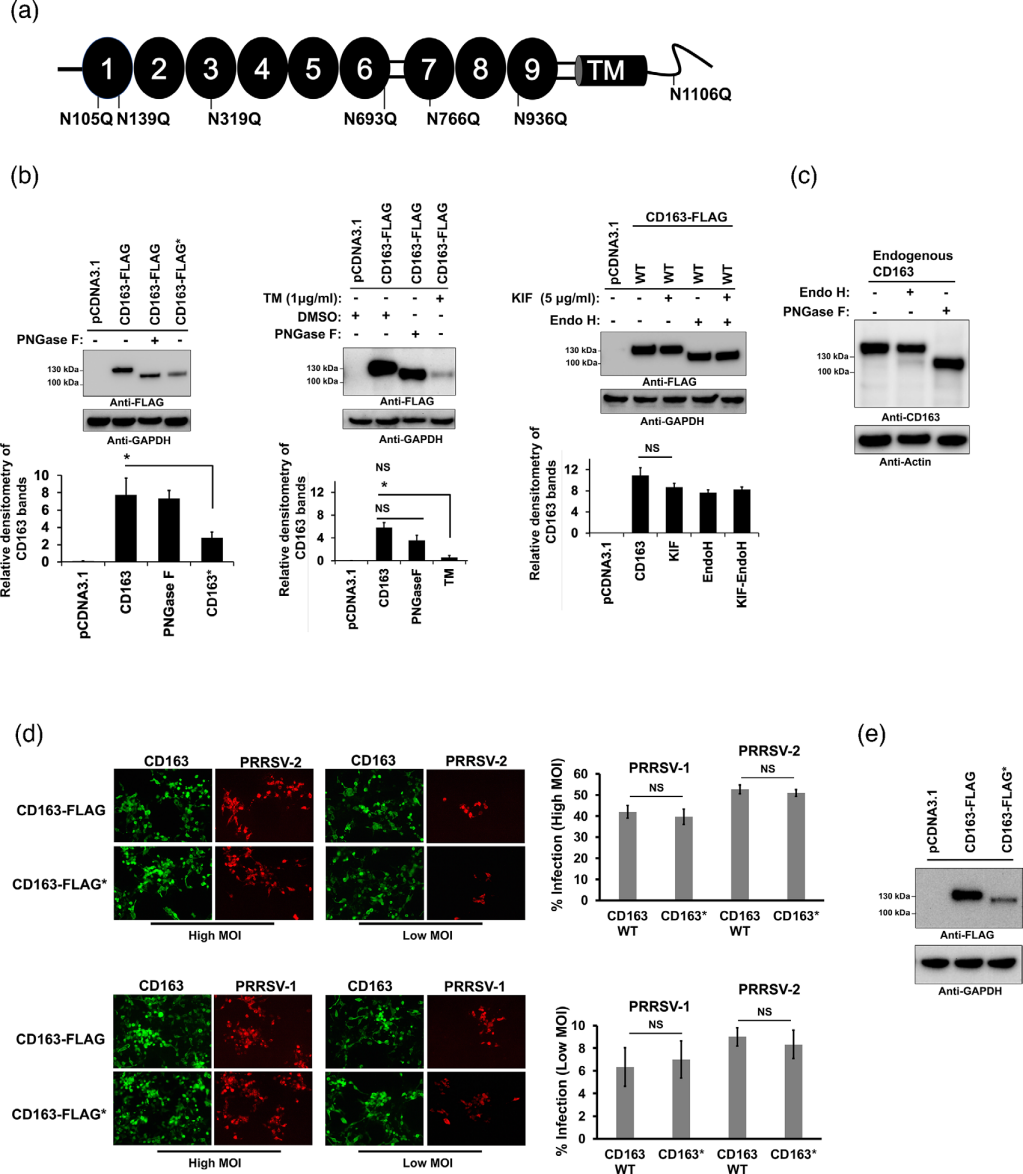
Effect of N-glycosylation inhibition of CD163 on PRRSV infection. (**a**) Schematic representation of the N-glycosylation sites of porcine CD163. Ovals and squares identify the SRCR and PST domains, respectively. The cylinder is the transmembrane domain. Seven potential N-glycosylation sites in CD163 are indicated. The N-glycosylation sites were predicted with the NetNGlyc-1.0 server [94]. (**b**) Left panels. HEK293T cells were transfected with plasmids expressing either CD163-FLAG or a N-glycosylation-deficient CD163 (CD163-FLAG*) and digested with PNGase F. CD163 was detected with anti-FLAG antibody. Middle panels. HEK293T cells were transfected with a plasmid expressing CD163 and treated with tunicamycin (TM) for 16 h before harvesting or digested with PNGase F. CD163 was detected with anti-FLAG antibody. Right panels. Western blots of CD163 expression levels after kifunensine (KIF) treatment for 16 h. Cell lysates were digested with Endo H and immunoblotted with anti-FLAG antibody. In all experiments, GAPDH was used as a loading control. Densitometry of CD163 bands is normalized to the loading control GAPDH. Fold changes are shown relative to empty vector (pCDNA3.1), and results are means±standard deviation (SD) values from three independent experiments (*, *P*<0.05; **, NS, not significant). CD163-FLAG* refers to N-glycosylation sites removed by replacing Asn with Gln. (**c**) Western blotting of CD163 expression levels in PAMs cells following N-glycosidases treatment. Cell lysates were digested with digested with Endo H or PNGase F and immunoblotted with anti-CD163 antibody. Bottom panel shows actin loading control. (**d**) Representative fluorescence images of PRRSV infectivity in HEK293T cells transiently expressing wild-type (WT) or the N-glycosylation-deficient CD163. HEK293T cells were transfected with plasmids expressing mutant or WT CD163 proteins. At 24 h post-transfection, the cells were infected with PRRSV-1 (Lelystad) or PRRSV-2 (VR2332) at MOI of 5 (high MOI) or 0.1 (low MOI). To visualize PRRSV infection, the infected cells were fixed and stained at 72 hpi as in Fig. S5. WT and mutant CD163 proteins were visualized by staining cells with anti-FLAG antibody and then with Alexa 488-goat anti-rabbit IgG (green). Percentages of infected cells for each CD163 variant are shown on the right. Results are means±standard deviation (SD) values from three independent experiments (NS, not significant). (**e**) Expression of the CD163 N-glycosylation mutant was analysed by Western blotting using anti-FLAG antibody and GAPDH as a loading control. Similar results were obtained in three independent experiments and representative data is shown.

### Removal or modification of N-glycans from PRRSV affect viral infection of PAMs

**Fig. 4.**
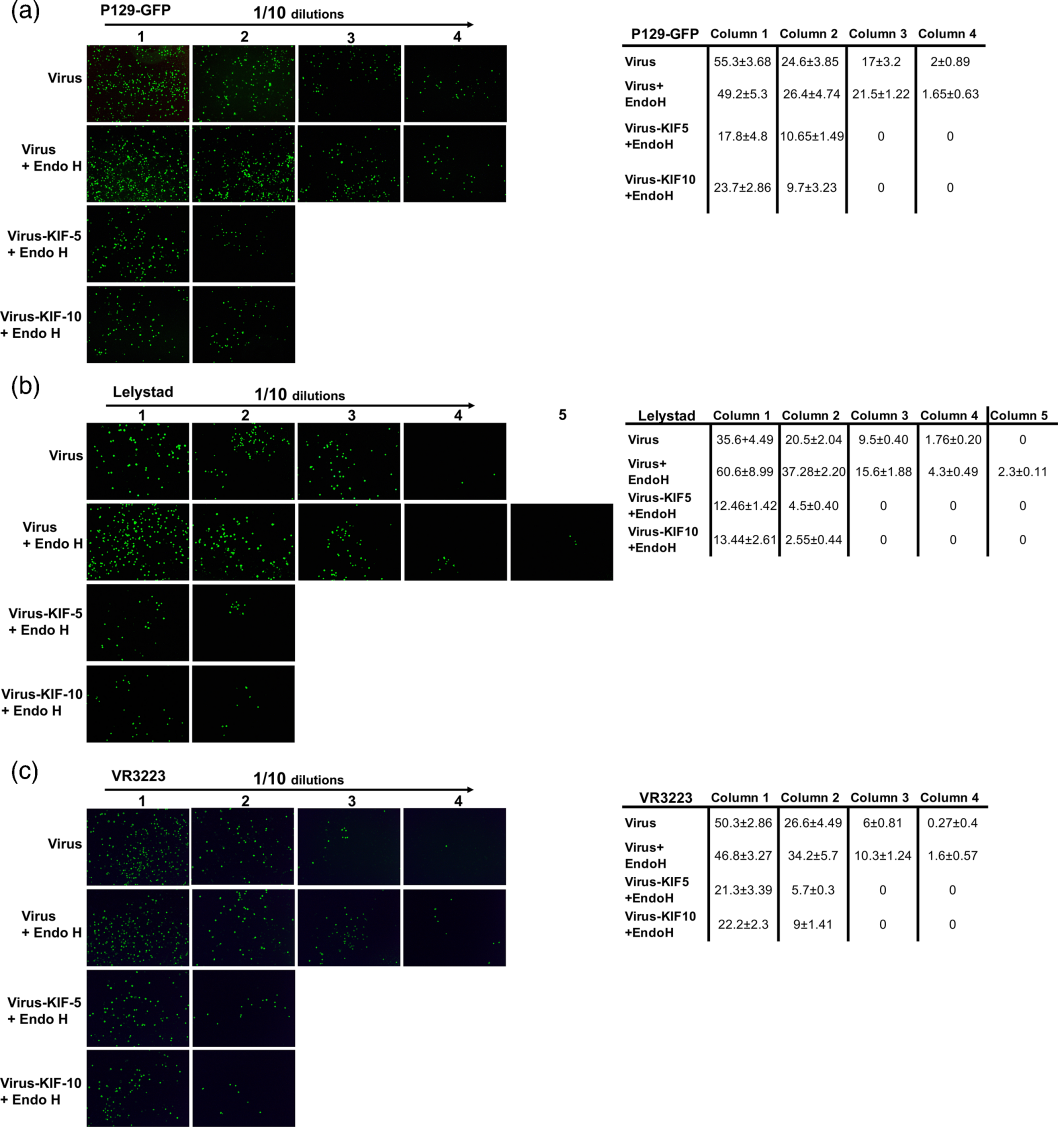
Effect of Endo H digestion on viral infection of PAMs. P129-GFP (**a**), Lelystad (**b**) and VR2332 (**c**) isolates propagated in absence or presence of KIF at 5–10 µg ml^−1^ in MARC-145 cells for 3 days were digested with Endo H at 37 °C for 1 h. Different dilutions of virus–enzyme mixtures (starting MOI=1) were inoculated onto PAMs at 37 °C for 1 h. After infection, cells were washed to remove enzyme and unbound virus, and further incubated for 24 h. Virus without enzyme treatment was incubated at 37 °C for 1 h to serve as a temperature stability control. To control for an enzyme effect on cells, Endo H incubated alone at 37 °C for 1 h was mixed with the temperature-stability control virus and the mixture was added to the cells. Cells were then fixed and stained as indicated Fig. 1. P129-GFP infection was visualized directly without antibodies. Similar results were obtained in three separate experiments and representative data is shown. The representative percentages of infected cells for each column are shown on the right. A positive result for infection was recorded as a cell expressing green fluorescence. Five to six fields were randomly selected in each sample to analyse 500–1000 individual cells. Results are means±standard deviation (SD) values from three independent experiments.

To further confirm the role of complex N-glycosylation on PRRSV infection, similar infection experiments described above were performed in primary cultures using the three PRRSV isolates utilized in this study. PRRSV harvested from MARC-145 cell cultures in the presence of 5 or 10 µg ml^−1^ of KIF were used to infect PAMs. As shown in Fig. 2, titration endpoint assays showed that complex-type N-glycans present in either of the PRRSV isolates are required for an optimal infection. The overall yields of the high mannose viruses were nearly one log less than that of WT viruses at 24 hpi. In addition, a drastic reduction in infection was detected after removing high mannose glycans from either of the KIF-propagated PRRSV isolates, indicating that the resulting monoglycosylated virions have lost infectivity in PAMs (Fig. 4a, b, c). Predictably, removing high-mannose and hybrid type N-glycans from PRRSV-1 by Endo H digestion increased the ability of PRRSV-1 to infect primary cultures by one log (Fig. 4b), confirming that PRRSV-1 and PRRSV-2 have different requirements of N-glycan structure for optimal infection. Overall, these results confirmed that the presence of high mannose type glycans on PRRSV surface is the minimal requirement for an efficient viral infection in PAMs.

### Removal of N-glycans from PRRSV affects viral entry but has no influence on viral attachment

**Fig. 5.**
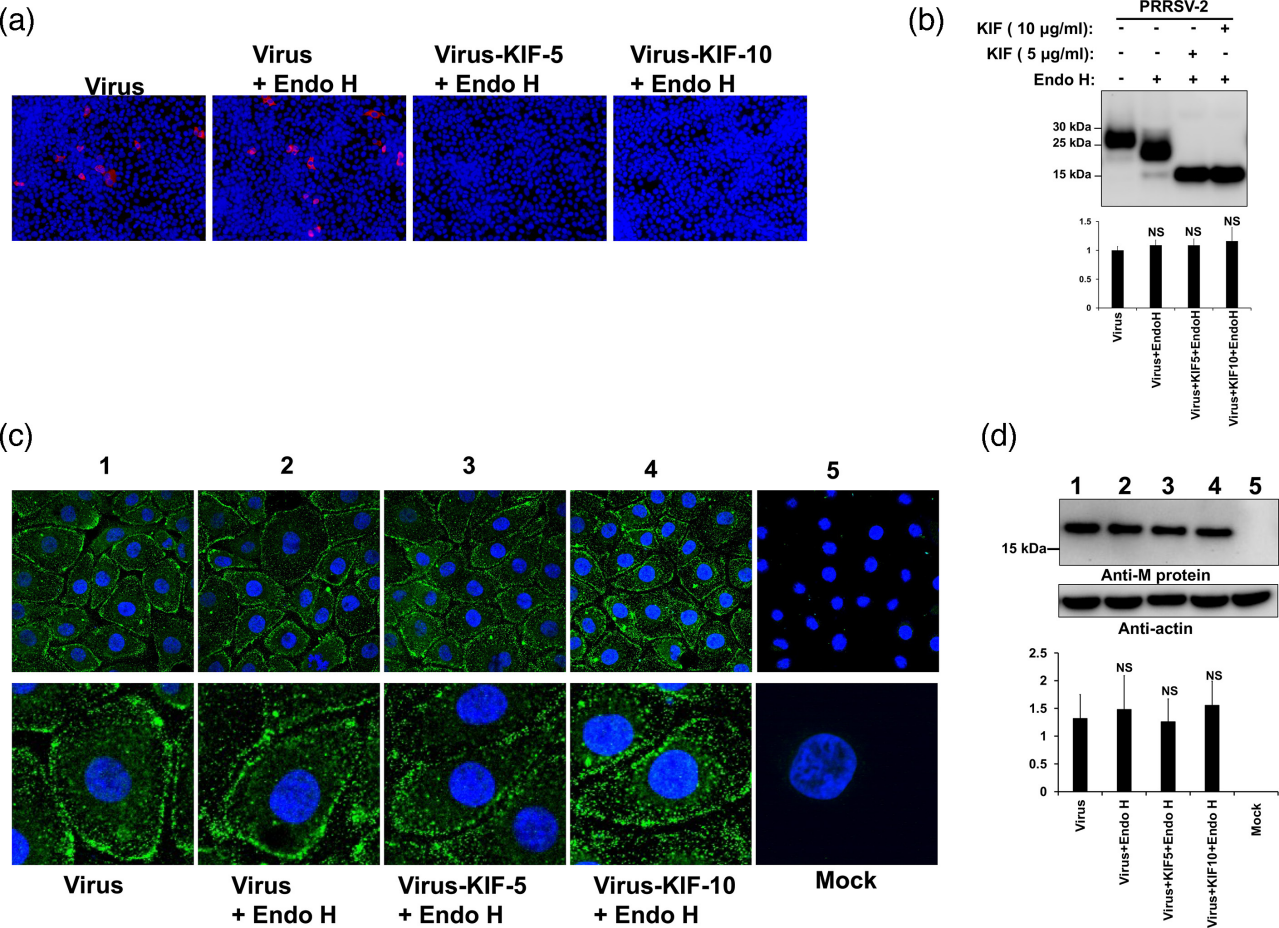
Effect of removal of N-glycans from PRRSV on viral entry and binding to the cell surface. (**a**) Effect of Endo H digestion on viral entry. PRRSV-2 (P129 strain) propagated in absence or presence of KIF at 5–10 µg ml^−1^ for 3 days was treated with Endo H at 37 °C for 1 h. Different virus–enzyme mixtures (MOI=1) were inoculated onto MARC-145 cells at 37 °C for 1 h. Cells were then washed to remove enzyme and unbound virus, and further incubated for 9 h. Virus without enzyme treatment was incubated at 37 °C for 1 h to serve as a temperature stability control. To control for an enzyme effect on cells, Endo H incubated alone at 37 °C for 1 h was mixed with the temperature-stability control virus and the mixture was added to the cells. Cells were then fixed and stained with PRRSV N protein antibody, followed by Alexa 594-goat anti-mouse IgG (red). Nuclei were counterstained with DAPI (blue). Similar results were obtained in three separate experiments and representative data is shown. (**b**) Western blot of GP5 expression levels of P129-GFP propagated in MARC-145 cells with KIF at 5–10 µg ml^−1^ and digested with Endo H. Proteins bands were detected with an anti-GP5 antibody. Densitometry of GP5 bands is shown below the immunoblot. Fold changes are shown relative to lane 1 (virus) and data are mean values± standard deviation (SD) of three independent experiments (NS, not significant). (**c**) P129-GFP was propagated in absence or presence of KIF and treated with Endo H as indicated above. Virus–enzyme mixtures (MOI=1) were inoculated onto MARC-145 cells at 4 °C for 1 h and subsequently washed to remove unbound virus. Virus without enzyme treatment was incubated at 37 °C for 1 h to serve as a temperature stability control. To control for an enzyme effect on cells, Endo H incubated alone at 37 °C for 1 h was mixed with the temperature-stability control virus and the mixture was added to the cells. Cells were then fixed, and stained with PRRSV N protein antibody, followed by Alexa 488-goat anti-mouse IgG (green). Nuclei were counterstained with DAPI (blue). Representative zoom images are shown on the bottom. Similar results were obtained in three separate experiments and representative data are shown. (**d**) The same as C, but after incubation of the virus–enzyme mixtures onto MARC-145 cells and subsequently washing to remove unbound virus, cells were lysed. Cell lysates were analysed with Western blotting and probed with antibodies directed against either the M protein or actin. Densitometry of M protein bands shown below the immunoblots are normalized to the loading control actin. Fold changes are shown relative to Lane one and data are mean values of three independent experiments (NS, not significant). Lane 1, Virus; Lane 2, Virus +Endo H; Lane 3, Virus +KIF-5+Endo H ; Lane 4, Virus +KIF-10+Endo H; Lane 5, Mock.

Previous studies reported that sialic acid-containing complex glycans are implicated in mediating virus attachment and internalization [35,36]. To evaluate the role of N-glycans on PRRSV viral entry, we performed a viral entry assay using monoglycosylated virions as previously described [51]. To this end, KIF-treated viruses digested with Endo H (Fig. 5b) were inoculated onto MARC-145 cells and the viral entry was visualized by immunostaining of newly synthesized viral N protein. Since one replication cycle of PRRSV takes approximately 12 h [51,52], infected cells were fixed at 10 h post-infection to assure that immunostained cells were an outcome of infection with the input virus and not by secondary spread. Results showed that removal of N-glycans affects the ability of PRRSV to enter the cell, since no infection was detected in KIF-treated viruses digested with Endo H (Fig. 5a). In contrast, infection was observed in cells infected with viruses digested with Endo H, which confirmed that only complex-type N-glycans present in PRRSV are required for cell entry (Fig. 5a). Next, to analyse whether complex or hybrid-type N-glycans of PRRSV are required for virus binding to the cell surface, a viral attachment assay was performed as previously described [66]. For this purpose, KIF-treated viruses digested with Endo H (Fig. 5b) were inoculated onto MARC-145 cells and the binding process was visualized by confocal microscopy. As shown in Fig. 5c, intact or digested viral particles clearly attached to the cell surfaces, suggesting that removal of complex N-glycans did not prevent virus adsorption to cell cultures. Similar results were obtained when we repeated the same binding assay with the VR2323 isolate of PRRSV-2 (Fig. S9). Next, we examined the viral binding by Western blot using a specific antibody that recognizes the viral envelope M protein. Following incubation of the different virus particles onto MARC-145 cells, whole-cell lysates were assessed via immunoblot analysis. Our results revealed comparable M protein levels in all samples, suggesting that a similar amount of viral particles were attached to the cell cultures (Fig. 5d).

### N-glycosylation of the viral envelope glycoproteins GP2, GP3, GP4 and GP5 is not necessary for the association to CD163

**Fig. 6.**
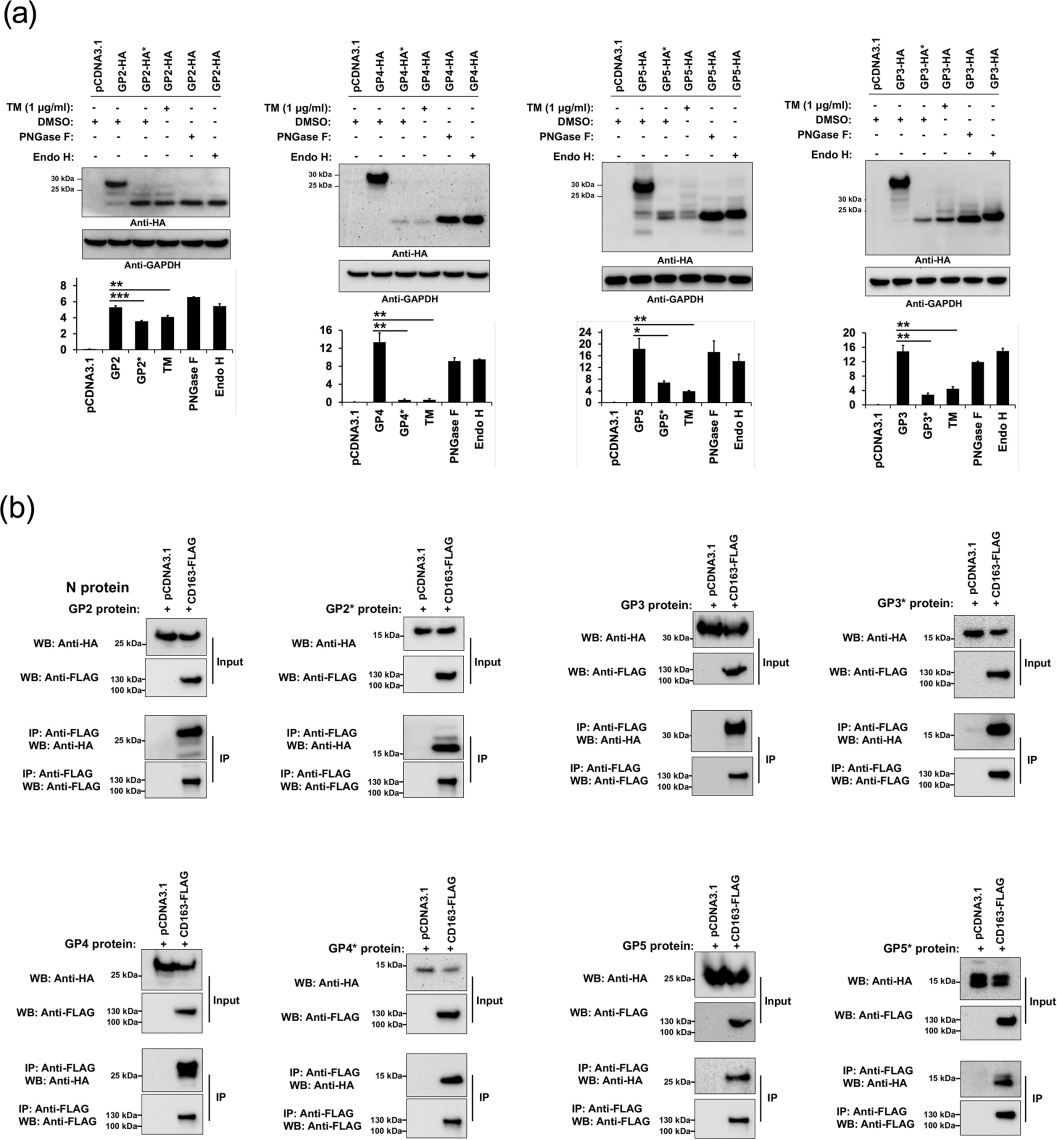
Blocking of N-glycosylation of the viral envelope glycoproteins does not disrupt their binding to CD163. (**a**) Western blotting of N-glycosylation-deficient viral glycoproteins variants expression levels in transfected cells. HEK293T cells were transfected with plasmids expressing GP2, GP3, GP4 and GP5 and treated with TM for 16 h before harvesting or digested with PNGase F or Endo H. Expression profiles of the N-glycosyaltion mutants are also shown. In all experiments, GAPDH was used as a loading control. Densitometry of viral glycoprotein bands are normalized to the loading control GAPDH. Fold changes are shown relative to empty vector (pCDNA3.1), and results are means±standard deviation (SD) values from three independent experiments (*, *P*<0.05; **, *P*<0.005; ***, *P*<0.0005; NS, not significant). (**b**) Interaction of CD163 with N-glycosylation-deficient PRRSV glycoproteins mutants. HEK293T cells were cotransfected with plasmids encoding HA-tagged individual viral glycoproteins or their N-glycosylation variants, and a plasmid expressing CD163-FLAG protein. Cells were lysed 24 h after transfection and analysed by Western blotting using anti-HA and anti-FLAG antibodies (Input). Subsequently, lysates were immunoprecipitated by using anti-FLAG agarose beads, as described in Methods. To control for background binding of the viral envelope proteins to anti-FLAG beads, we performed similar experiments with HEK293T cells that were cotransfected with plasmids encoding HA-tagged individual viral glycoproteins variants and an empty pCDNA3.1 vector. Anti-FLAG agarose beads were eluted using FLAG peptide, and elutions were analysed by Western blotting using anti-HA and anti-FLAG antibodies (Immunoprecipitation). Similar results were obtained in three independent experiments and representative data is shown. WB, Western blot; IP, Immunoprecipitation.

**Fig. 7.**
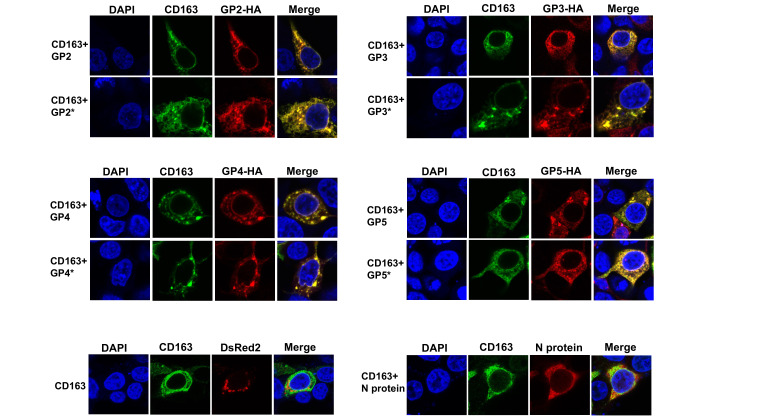
Cellular colocalization of CD163 with N-glycosylation-deficient PRRSV glycoproteins. Semiconfluent monolayers of HEK 293 T cells were cotransfected with a plasmid expressing CD163-FLAG and plasmids encoding either wild-type or mutant HA-tagged individual viral glycoproteins. At 24 h post-transfection, cells were fixed and immunostained using anti-FLAG antibody followed by Alexa 488-conjugated goat anti-rabbit IgG (green). The viral glycoproteins were visualized using anti-HA monoclonal antibody, followed by Alexa 594-goat anti-mouse IgG (red). Nuclei were counterstained with DAPI (blue). Representative colocalization (yellow) images are shown. Similar results were obtained in three separate experiments and representative data are shown. As negative controls, cells were cotransfected with either a DsREd-Mito-expressing plasmid or a plasmid expressing the N-protein and a plasmid expressing CD163-FLAG. At 24 h post-transfection, cells were fixed and immunostained as indicated above. Mitochondria were visualized directly without antibodies (red). N protein was immunostained as indicated Fig. 1. Nuclei were counterstained with DAPI (blue). Similar results were obtained in three separate experiments and representative data are shown.

Previous work has showed that the viral envelope proteins GP2, GP3, GP4 and GP5 specifically interact with CD163 [31,34]. Additionally, another study demonstrated that N-glycosylation of GP2 and GP4 is required for efficient interaction with CD163 [27]. To analyse the role of N-glycosylation of the viral GPs on the interaction with CD163, we generated N-glycosylation-deficient mutants in which all the N present in the consensus N-glycosylation sites were replaced by Q (for clarity, these new mutants are referred to as GP2*, GP3*, GP4* and GP5*). To confirm that the viral GP variants were N-glycosylation null mutants, we analysed their expression and electrophoretic mobility after tunicamycin (TM) treatment in transfected cells. Additionally, digestion of viral GPs with PNGase F, which cleaves all N-glycans, was performed. As expected, expression of N-glycosylation-deficient mutants resulted in detection of bands with a molecular weight similar to that of TM-treated lysates or samples digested with PNGase F (Fig. 6a). Interestingly, TM treatment showed reduced levels of all the N-glycosylation variants (Fig. 6a). The effect of TM was mimicked by the different mutant constructs, which lacked glycans (Fig. 6a). Together, these data suggest that N-glycosylation is important for proper protein folding and stability of the viral GPs. Digestion of cell lysates with Endo H, resulted in protein bands with molecular weight similar in size to the N-glycosylation-deficient mutants, suggesting that the N-glycans present on the viral GPs expressed individually in transfected cells are high-mannose or hybrid-type glycans (Fig. 6a). Next, to assess the importance of N-glycosylation of the viral GPs on their association with CD163, we analysed the biochemical ability of CD163 containing a FLAG tag to interact with the different viral GP variants containing an HA tag. To this end, we co-transfected similar amounts of plasmid expressing CD163-FLAG with plasmids expressing each of the four viral GPs mutants. Cells were lysed and FLAG-tagged CD163 variants were immunoprecipitated by using anti-FLAG beads. Proteins eluted by the FLAG peptide were separated by SDS-PAGE gels and analysed by Western blotting using anti-HA and anti-FLAG antibodies (Fig. 6b). Surprisingly, in contrast with a previous report, which showed that glycosylation of GP2 and GP4 proteins is required for an efficient interaction with CD163 [27], under our experimental conditions, the different viral GP variants were efficiently coprecipitated by the anti-FLAG antibody (Fig. 6b). These results indicate that N-glycosylation is not required for the ability of the different viral GPs to bind CD163. Colocalization experiments in co-transfected cells confirmed the association of the CD163 with the viral glycoprotein mutants (Fig. 7). Overall, these findings indicated that N-glycosylation of the viral envelope GPs is not necessary for efficient interaction with the receptor CD163. To validate our colocalization studies, we included as a negative controls the mitochondrial marker dsRed2-Mito, that does not localize with CD163 [32], as well as the N-protein, which does not associate with CD163 [34]. As expected, CD163 did not colocalize with neither of the negative controls (Fig. 7).

## Discussion

The main purpose of this study was to examine the influence of N-glycosylation of PRRSV on viral infection. The approach used in this study was to investigate the effect of N-glycan perturbations to understand the role of PRRSV N-glycans in permissive cell infection. To this end, we have generated monoglycosylated PRRSV virions by using a strategy that consists of propagating PRRSV in presence of a glycosylation inhibitor followed by Endo H digestion, which leaves a single N-acetylglucosamine residue (GlcNAc) [67]. Digestion conditions were established by monitoring changes on the molecular weight of GP5, the most abundant envelope glycoprotein, by Western blot. Due to the lack of functional antibodies against GP2-4, we were not able to evaluate their N-glycosylation state after KIF and Endo H treatment. However, based on the substantial results obtained with GP5, it is likely that all or most of the minor surface glycoproteins were monoglycosylated by using the experimental approach described in this report. This approach was taken as opposed to removing all the glycans from PRRSV since the total removal of N-glycans from proteins can cause misfolding of the viral GPs. This strategy is generally used to obtain crystal structures of deglycosylated proteins while avoiding the complications of misfolding, as well as to study the role of N-glycosylation in protein-protein interactions [67,68]. Furthermore, the first GlcNAc of N-glycosylation is the most important glycan in terms of providing the most of the energy to stabilize the native state of the protein, and the minimum glycan component required for protein stability [69,70]. Thus, the production of monoglycosyalted virions in the current study is a rational design to trim down the glycan on the viral GPs to monoglycosylated GlcNAc without affecting the structural integrity of the virion. In agreement with this notion, we found that a complete removal of N-glycans of the viral GPs by mutagenesis or TM treatment induced a low expression of all the viral GPs in transfected cells, indicating that the blocking of N-glycosylation might affect the stability or biosynthesis of the viral envelope proteins (Fig. 6a). Consistent with previous reports, we verified, by using the monoglycosylated PRRSV production strategy, that GP5 incorporated into the PRRSV virion contains complex N-linked structures [24,51, 56]. The pattern observed after enzymatic digestion of viral particles with Endo H agrees with a previous mass spectrometric analysis of carbohydrates attached to GP5 of purified PRRSV-2, which revealed the presence of mostly complex-type N-glycans, but also a small fraction of Endo H sensitive N-glycans [56]. In contrast, GP5 expressed individually in transfected cells contains Endo H-sensitive high-mannose and hybrid type N-glycans (Fig. 6a). A similar N-glycosyaltion profile was described when GP5 was expressed in a similar way [24,51]. One likely explanation is that GP5 expressed in transfected cells might accumulate mostly in the endoplasmic reticulum (ER) or in the *cis*-Golgi region and therefore remains Endo H sensitive. In contrast, in infected cells, GP5 might interact with other viral proteins forming protein complexes that facility the transport of GP5 to the medial and/or *trans*-Golgi region where the majority of GP5 molecules acquire Endo H resistant N-glycans prior to being incorporated into PRRSV virions. GP5 forms a disulphide-linked heterodimer complex with M protein [20]. Such association may occur prior to further processing of N-linked side chains, probably before GP5 is carried out of the ER or the *cis*-Golgi compartment. In agreement with this possibility, it was previously reported that co-expression of both viral proteins generated a small fraction of GP5 resistant to Endo H, indicating that a small fraction of GP5 molecules was transported to the medial-Golgi or further when co-expressed with M protein [51,71].

A remarkable observation in our experiments was that removal of the PRRSV complex-type N-glycans slightly affected infection. Additionally, we found that the presence of high mannose type glycans on either PRRSV-1 or PRRSV-2 surface is the minimal requirement for an efficient viral infection. Finally, we demonstrated that a complete depletion of high mannose type glycans from KIF-propagated virus drastically blocked infection in MARC-145 cells. In contrast, a previous report showed that PNGase F treatment of PRRSV-2 (VR2332) did not alter viral infection in MARC-145 cells [38]. However, different lines of evidence support our conclusion regarding the importance of complex N-glycosylation in PRRSV infection. First, we confirmed by using the PRRSV-1 prototype strain, Lelystad virus, the PRRSV-2 prototype strain, VR-2332, and a highly pathogenic PRRSV-2 strain, P129, that complex N-glycans are required for a productive infection by generating virus containing high mannose type N-glycans. Second, monoglycosylated viruses produced after Endo H digestion of high mannose-containing viral particles lost the ability to efficiently infect permissive cells. These results are in agreement with previous findings that showed that PNGase F treatment of type 1 PRRSV reduced infection in PAMs [35]. However, in contrast to this report that also showed that complex-type N-glycans are essential for PRRSV-1 infection, we found that the presence of high mannose type glycans on either PRRSV-1 or PRRSV-2 surface is the minimal requirement for a productive viral infection. Although our observations also indicated that removal of complex N-glycans slightly affected viral infection. Third, in agreement with our data, numerous reverse genetics experiments demonstrated the importance of specific N-glycosylation sites of the minor envelope proteins, GP2-4, and of GP5 for viral infectivity [19,24, 26,28, 72]. Although the negative impact in viral infection after removing specific N-glycan residues of the viral envelope proteins could be attributed to the fact that glycans in these sites are important for proper protein folding and stability, and thus for virus assembly. In agreement with this notion, our transfection experiments showed that the N-glycosylation-deficient GPs mutants have a low-level expression, indicating that the blocking of N-glycosylation might affect the proper folding or biosynthesis of the viral envelope proteins. Another possible explanation for the discrepancies in the PRRSV infection results is that, although the same PRRSV-2 VR2332 strain was used in both studies [38], variations among isolates within PRRSV-2 VR2332 strain could produce different outcomes for the infection experiments. Indeed, consistent with this idea, previous studies showed that replacement of the N-glycosylation site N44 of GP5 with alanine residue produced viruses that were not viable in cell culture [24]. In contrast, when the N44 was substituted with K, a viable virus was generated [72], indicating that mutants with deletion of a glycan in GP5 display different growth efficiencies in permissive cells depending on the amino acid substitutions at N-glycosylation sites.

Consistent with results observed in MARC-145 cells, infection of PAMs was also affected by either removing complex N-glycans or high mannose glycans structures from KIF treated viruses. Although, the level of infection in PAMs was higher than in MARC-145 cells, suggesting that PAMs are more susceptible to be infected with monoglycosylated PRRSV virions. These results agree with a previous report showing that PNGase F treatment of PRRSV-1 produced a drastic reduction in viral infection in PAMs, whereas enzymatic treatment of PAMs had no effect [35]. However, a more recent report showed that PAMs incubation with either PNGase F or Endo H significantly reduced PRRSV-2 infection equivalently to the reduction caused by endoglycosidases digestion of the virus. Therefore, they concluded that the inhibitory effect was not specific to the virus since endoglycosidase treatment of PAMs before addition of viral particles also significantly reduced infection. In the same report, it was suggested that the negative impact on infection could be due to modifications of the glycan structure of cell surface viral receptor, CD163. In contrast to these observations, we found that incubation of PAMs with virus–enzyme mixtures containing six times more Endo H than in the previous report did not have a negative impact on viral infection. In addition, here, we show that removal of the N-glycosylation sites, by mutagenesis of CD163 did not disrupt its ability to support viral infection in non-permissive transfected cells. Interestingly, we verified, by digestion with glycosidases and by treatment with inhibitors that interfere at different stages of N-glycosylation biosynthesis, that porcine CD163 is N-linked glycosylated. De-N-glycosylation enzymatic experiments revealed the presence of high mannose/hybrid and complex type glycans in endogenous CD163. On the other hand, digestion with Endo H revealed the presence of high mannose/hybrid type glycans in CD163, when the viral receptor was exogenously expressed in non-permissive HEK293T cells. One possible explanation for these discrepancies is that CD163 expressed in transfected cells might accumulate mostly in the endoplasmic reticulum (ER) or in the *cis*-Golgi region and therefore remains Endo H sensitive. In agreement with this notion, we have previously shown that in addition to localize in the cell surface, CD163 accumulates in the ER when was exogenously expressed in HEK293T cells [33]. In contrast, in PAMs, CD163 might be transported to the medial and/or *trans*-Golgi region where the majority of CD163 molecules acquire Endo H resistant complex N-glycans. Our findings also demonstrate that the complete deglycosylation of CD163 by site-directed mutagenesis or TM treatment results in a significant decrease of protein expression, which agrees with the fact that N-glycosylation is important for protein folding and stability [73,75].

Our infection experiments also showed that PRRSV-1 and PRRSV-2 have different requirements of N-glycan structures for an efficient infection. Removal of high mannose and hybrid type glycans from PRSV-1 virions increased viral infection in both MARC-145 and PAMs cells. Modification of N-glycosylation is a common mechanism used by viruses during the process of viral evolution to improve the survival and transmissibility of a virus [76]. For example, N-glycans of HIV glycoprotein120 are required to viral infection and transmission [77]. Most of the HIV N-glycan deficient mutants showed a reduction in infectivity, however two of the N-glycan mutants showed an increased in infectivity and transmission efficiency [77]. Similarly, adding an N-glycosylation site to the HA protein can increase the innate immune response in the respiratory system, and reduce the transmissibility of influenza A viruses [78,80]. In addition, specific mutations in the SARS-CoV-2 virus spike glycoprotein can significantly reduce the infectivity of SARS-CoV-2, suggesting that their glycans are important for viral infectivity [55,81,83]. Previous work for Sindbis virus also showed that single glycosylation mutations in the E2 glycoprotein resulted in viruses with better replication characteristics than the WT virus [84]. For PRRSV, by using reverse genetics technology it has been shown that infectious clones possessing deletions in individual N-glycosylation sites of GP4 had higher litres than the WT virus. The enhancement of virus replication in GP4 mutated viruses could not be explained [27]. In line with our observations, all these findings indicate that alteration of the N-glycosylation pattern in viruses can improve or decline viral infectivity. One possible explanation for the increase in viral infection of PRRSV-1 after removal of high mannose and hybrid type glycans from virions, is that the GPs fold in a manner that is more favourable for viral infection as compared to the parental virus. This might potentially be achieved through increased interactions among the viral GPs and/or interactions with other host cell molecules that are implicated in PRRSV entry and replication. Further studies will be needed to determine how removal of high mannose and hybrid type glycans from PRRSV-1 enhance virus infectivity.

Our virus yield assays in MARC-145 cells revealed that there was no difference in viral growth curves at 72 hpi for PRRSV-2 isolates propagated at KIF concentrations of 10 µg ml^−1^. However, a similar effect was only observed for PRRSV-1 at KIF concentrations of 5 µg ml^−1^. Previous experiments showed that cells treated with KIF exhibited significantly higher permeability than untreated cells. A dose-dependent effect was observed with KIF, which controlled the extent of conversion of all cell surface N-glycans into high mannose type glycans [85]. In addition, it has been shown that increasing concentrations of KIF changed the composition of high-mannose glycoforms of recombinant human acid α-glucosidase, which is a therapeutic enzyme for Pompe disease [86]. Therefore, we speculate that the differences on virus yield between PRSSV genotypes propagated at different concentrations of KIF could be attributed to changes in the levels of high mannose glycoforms of the viral surface, which would induce conformational changes on the viral envelope glycoproteins affecting the viral infectivity. Consistent with this idea, our data revealed that modification of N-glycan structure of PRRSV-1 by digestion with Endo H increases the capacity of the virus to infect permissive cells.

To analyse the role of N-glycosylation in PRRSV, different genetic ablation experiments have been performed. The N-glycosylation sites N184 of GP2, N42, N50 and N131 of GP3, multiple N-sites (N37, 84, 120 and 130) of GP4, and N44 of GP5 are required for PRRSV infection [24,27,29, 72], suggesting that glycans in these sites are critical for proper protein folding and stability, and thus for virus assembly. Deficiencies in protein folding and stability, intracellular trafficking, virion assembly, or egress have been observed for different enveloped viruses including influenza virus, Dengue virus, Japanese encephalitis virus and Hantaan virus, when their envelope GPs were mutated at specific N-glycosylation sites [87]. Consistent with these evidences, our data showed that a complete depletion of N-glycosylation sites from the viral GPs, induced a low expression of all the viral GPs in transfected cells, suggesting that the blocking of N-glycosylation affects the stability or expression of the viral envelope proteins. However, the role of N-glycosylation of the minor envelope proteins for viral infectivity is not clear. Previous reports showed that mutations in the two predicted N-glycan sites of GP2 had no effect on viral infectivity [26,28]. On the other hand, other study suggested that viral interaction with CD163 was reduced after removing all N-glycosylation sites, although single mutations in either site had no effect on the association with CD163 [27], despite the same report showed that N184 of GP2 is required for viral infection [27].

In contrast to a previous study that showed that complex N-glycosylation is involved in PRRSV attachment [35], our binding experiments showed that monoglycosylated virions lacking complex and hybrid-glycans did not lose the capacity to bind to the cell surface. This observation is reminiscent of the results with SARS-CoV-2, in which removal of the N-glycosylation sites from the envelope Spike glycoprotein had only minor contribution to Spike-receptor binding [47,55]. However, these carbohydrates played a major role in regulating viral entry [55]. In contrast, other studies have reported for other viruses an important role of the sialic acid present in the complex N-glycans on ligand-receptor interactions, including the coronavirus, such as the Middle East respiratory syndrome virus/MERS [88], parvovirus [89], and influenza [90,91].

A previous report showed that glycosylation of GP2 and GP4 proteins is required for an efficient interaction with CD163 [27]. In contrast, our colocalization and immunoprecipitation assays to evaluate the role of N-glycosylation of the GPs in the association with CD163 demonstrated that removal of all N-glycans on GPs did not prevent binding to CD163, suggesting that glycans do not play a critical role in forming the interaction between PRRSV GPs and CD163. Previous reports have demonstrated that the viral glycoproteins GP2/GP3/GP4 form a viral heterotrimer while GP5 and M protein form heterodimer complexes during PRRSV infection [13]. Therefore, our immunoprecipitation and co-localization experiments, which examined the ability of the viral receptor to interact with individual viral envelope proteins, cannot exclude the possibility that mutations in a viral glycoprotein may impact the CD163-viral glycoprotein association when the viral envelope proteins form dimeric or trimeric complexes during infection. However, our binding experiments displayed in Fig. 5 showing that monoglycosylated virions lacking complex and hybrid-glycans did not lose the capacity to bind to the cell surface, aligns with our immunoprecipitation and colocalization experiments with individual viral glycoproteins. In agreement with the notion that N-glycosylation of viral envelope proteins are not required for binding to the cellular receptor, binding studies revealed that N-glycosylation is not required for the interaction of the Spike protein of SARS-COV-2 with its receptor ACE2 [47,55]. In contrast, there are several examples for the requirement of N-glycosylation in the formation of protein-protein interactions. For instance, N-glycosylation of the vasopressin V1a receptor is required for optimal receptor-ligand binding [92] and N-glycosylation of P2Y12 receptor is necessary to trigger proper downstream Gi-mediated signalling [93].

In conclusion, by using a strategy to produce monoglycosylated PRRSV, we found that the presence of N-glycans in the viral GPs appear to be required for an efficient infection of permissive cells. Additionally, our findings provide important information for understanding PRRSV infection and increase our knowledge of viral pathogenesis.

## supplementary material

10.1099/jgv.0.001994 Uncited Supplementary Material 1.

## Abbreviations

GPs: glycoproteins
KIF: kifunensine
PAMs: porcine alveolar macrophages
PRRSV: porcine reproductive and respiratory syndrome virus
TM: tunicamycin

## References

[1] Snijder EJ, Meulenberg JJ (1998). The molecular biology of arteriviruses. J Gen Virol.

[2] Cavanagh D (1997). Nidovirales: a new order comprising Coronaviridae and Arteriviridae. Arch Virol.

[3] Adams MJ, Lefkowitz EJ, King AMQ, Harrach B, Harrison RL (2017). Changes to taxonomy and the International Code of Virus Classification and Nomenclature ratified by the International Committee on Taxonomy of Viruses (2017). Arch Virol.

[4] Brinton MA, Gulyaeva AA, Balasuriya UBR, Dunowska M, Faaberg KS (2021). ICTV Virus Taxonomy Profile: Arteriviridae 2021. J Gen Virol.

[5] Kuhn JH, Lauck M, Bailey AL, Shchetinin AM, Vishnevskaya TV (2016). Reorganization and expansion of the nidoviral family Arteriviridae. Arch Virol.

[6] Holtkamp DJ, Kliebenstein JB, Neumann E, Zimmerman JJ, Rotto H (2013). Assessment of the economic impact of porcine reproductive and respiratory syndrome virus on United States pork producers. J Swine Health Prod.

[7] Neumann EJ, Kliebenstein JB, Johnson CD, Mabry JW, Bush EJ (2005). Assessment of the economic impact of porcine reproductive and respiratory syndrome on swine production in the United States. J Am Vet Med Assoc.

[8] Renken C, Nathues C, Swam H, Fiebig K, Weiss C (2021). Application of an economic calculator to determine the cost of porcine reproductive and respiratory syndrome at farm-level in 21 pig herds in Germany. Porcine Health Manag.

[9] Chae C (2021). Commercial PRRS modified-live virus vaccines. Vaccines.

[10] Duan X, Nauwynck HJ, Pensaert MB (1997). Virus quantification and identification of cellular targets in the lungs and lymphoid tissues of pigs at different time intervals after inoculation with porcine reproductive and respiratory syndrome virus (PRRSV). Vet Microbiol.

[11] Law SKA, Micklem KJ, Shaw JM, Zhang X, Dong Y (1993). A new macrophage differentiation antigen which is a member of the scavenger receptor superfamily. Eur J Immunol.

[12] Ritter M, Buechler C, Langmann T, Schmitz G (1999). Genomic organization and chromosomal localization of the human CD163 (M130) gene: a member of the scavenger receptor cysteine-rich superfamily. Biochem Biophys Res Commun.

[13] Su C-M, Rowland RRR, Yoo D (2021). Recent advances in PRRS virus receptors and the targeting of receptor-ligand for control. Vaccines (Basel).

[14] Ye N, Wang B, Feng W, Tang D, Zeng Z (2022). PRRS virus receptors and an alternative pathway for viral invasion. Virus Res.

[15] Li R, Qiao S, Zhang G (2022). Reappraising host cellular factors involved in attachment and entry to develop antiviral strategies against porcine reproductive and respiratory syndrome virus. Front Microbiol.

[16] Kappes MA, Faaberg KS (2015). PRRSV structure, replication and recombination: origin of phenotype and genotype diversity. Virology.

[17] Lunney JK, Fang Y, Ladinig A, Chen N, Li Y (2016). Porcine Reproductive and Respiratory Syndrome Virus (PRRSV): pathogenesis and interaction with the immune system. Annu Rev Anim Biosci.

[18] Han M, Yoo D (2014). Engineering the PRRS virus genome: updates and perspectives. Vet Microbiol.

[19] Stoian AMM, Rowland RRR (2019). Challenges for Porcine Reproductive and Respiratory Syndrome (PRRS) vaccine design: reviewing virus glycoprotein interactions with CD163 and targets of virus neutralization. Vet Sci.

[20] Veit M, Matczuk AK, Sinhadri BC, Krause E, Thaa B (2014). Membrane proteins of arterivirus particles: structure, topology, processing and function. Virus Res.

[21] Kappes MA, Miller CL, Faaberg KS (2013). Highly divergent strains of porcine reproductive and respiratory syndrome virus incorporate multiple isoforms of nonstructural protein 2 into virions. J Virol.

[22] Spilman MS, Welbon C, Nelson E, Dokland T (2009). Cryo-electron tomography of porcine reproductive and respiratory syndrome virus: organization of the nucleocapsid. J Gen Virol.

[23] Vu HLX, Kwon B, Yoon K-J, Laegreid WW, Pattnaik AK (2011). Immune evasion of porcine reproductive and respiratory syndrome virus through glycan shielding involves both glycoprotein 5 as well as glycoprotein 3. J Virol.

[24] Ansari IH, Kwon B, Osorio FA, Pattnaik AK (2006). Influence of N-linked glycosylation of porcine reproductive and respiratory syndrome virus GP5 on virus infectivity, antigenicity, and ability to induce neutralizing antibodies. J Virol.

[25] Popescu LN, Trible BR, Chen N, Rowland RRR (2017). GP5 of porcine reproductive and respiratory syndrome virus (PRRSV) as a target for homologous and broadly neutralizing antibodies. Vet Microbiol.

[26] Wissink EHJ, Kroese MV, Maneschijn-Bonsing JG, Meulenberg JJM, van Rijn PA (2004). Significance of the oligosaccharides of the porcine reproductive and respiratory syndrome virus glycoproteins GP2a and GP5 for infectious virus production. J Gen Virol.

[27] Das PB, Vu HLX, Dinh PX, Cooney JL, Kwon B (2011). Glycosylation of minor envelope glycoproteins of porcine reproductive and respiratory syndrome virus in infectious virus recovery, receptor interaction, and immune response. Virology.

[28] Wei Z, Tian D, Sun L, Lin T, Gao F (2012). Influence of N-linked glycosylation of minor proteins of porcine reproductive and respiratory syndrome virus on infectious virus recovery and receptor interaction. Virology.

[29] Wissink EHJ, Kroese MV, van Wijk HAR, Rijsewijk FAM, Meulenberg JJM (2005). Envelope protein requirements for the assembly of infectious virions of porcine reproductive and respiratory syndrome virus. J Virol.

[30] Lee C, Bachand A, Murtaugh MP, Yoo D (2004). Differential host cell gene expression regulated by the porcine reproductive and respiratory syndrome virus GP4 and GP5 glycoproteins. Vet Immunol Immunopathol.

[31] Das PB, Dinh PX, Ansari IH, de Lima M, Osorio FA (2010). The minor envelope glycoproteins GP2a and GP4 of porcine reproductive and respiratory syndrome virus interact with the receptor CD163. J Virol.

[32] Stoian AMM, Rowland RRR, Brandariz-Nuñez A (2022). Identification of CD163 regions that are required for porcine reproductive and respiratory syndrome virus (PRRSV) infection but not for binding to viral envelope glycoproteins. Virology.

[33] Stoian AMM, Rowland RRR, Brandariz-Nuñez A (2022). Mutations within scavenger receptor cysteine-rich (SRCR) protein domain 5 of porcine CD163 involved in infection with porcine reproductive and respiratory syndrome virus (PRRS). J Gen Virol.

[34] Yu P, Wei R, Dong W, Zhu Z, Zhang X (2020). CD163ΔSRCR5 MARC-145 Cells Resist PRRSV-2 Infection via Inhibiting Virus Uncoating, Which Requires the Interaction of CD163 With Calpain 1. Front Microbiol.

[35] Delputte PL, Nauwynck HJ (2004). Porcine arterivirus infection of alveolar macrophages is mediated by sialic acid on the virus. J Virol.

[36] Van Breedam W, Van Gorp H, Zhang JQ, Crocker PR, Delputte PL (2010). The M/GP(5) glycoprotein complex of porcine reproductive and respiratory syndrome virus binds the sialoadhesin receptor in a sialic acid-dependent manner. PLoS Pathog.

[37] Van Gorp H, Van Breedam W, Delputte PL, Nauwynck HJ (2008). Sialoadhesin and CD163 join forces during entry of the porcine reproductive and respiratory syndrome virus. J Gen Virol.

[38] Li J, Murtaugh MP (2015). Functional analysis of porcine reproductive and respiratory syndrome virus N-glycans in infection of permissive cells. Virology.

[39] Yoo D, Welch S-K, Lee C, Calvert JG (2004). Infectious cDNA clones of porcine reproductive and respiratory syndrome virus and their potential as vaccine vectors. Vet Immunol Immunopathol.

[40] Pei Y, Hodgins DC, Wu J, Welch S-KW, Calvert JG (2009). Porcine reproductive and respiratory syndrome virus as a vector: immunogenicity of green fluorescent protein and porcine circovirus type 2 capsid expressed from dedicated subgenomic RNAs. Virology.

[41] Meulenberg JJ, Hulst MM, de Meijer EJ, Moonen PL, den Besten A (1993). Lelystad virus, the causative agent of porcine epidemic abortion and respiratory syndrome (PEARS), is related to LDV and EAV. Virology.

[42] Benfield DA, Nelson E, Collins JE, Harris L, Goyal SM (1992). Characterization of swine infertility and respiratory syndrome (SIRS) virus (isolate ATCC VR-2332). J Vet Diagn Invest.

[43] Cafruny WA, Duman RG, Rowland RR, Nelson EA, Wong GH (2008). Antibiotic-mediated inhibition of Porcine Reproductive and Respiratory Syndrome Virus (PRRSV) infection: a novel quinolone function which potentiates the antiviral cytokine response in MARC-145 cells and pig macrophages. Virol Res Treat.

[44] Lee C, Calvert JG, Welch S-K, Yoo D (2005). A DNA-launched reverse genetics system for porcine reproductive and respiratory syndrome virus reveals that homodimerization of the nucleocapsid protein is essential for virus infectivity. Virology.

[45] Guo R, Katz BB, Tomich JM, Gallagher T, Fang Y (2016). Porcine reproductive and respiratory syndrome virus utilizes nanotubes for intercellular spread. J Virol.

[46] Guo R, Yan X, Li Y, Cui J, Misra S (2021). A swine arterivirus deubiquitinase stabilizes two major envelope proteins and promotes production of viral progeny. PLoS Pathog.

[47] Rowland R, Brandariz-Nuñez A (2021). Analysis of the role of N-linked glycosylation in cell surface expression, function, and binding properties of SARS-CoV-2 receptor ACE2. Microbiol Spectr.

[48] Lj R (1938). A simple method of estimating fifty per cent endpoints. Am J Hyg.

[49] Wells KD, Bardot R, Whitworth KM, Trible BR, Fang Y (2017). Replacement of porcine CD163 scavenger receptor cysteine-rich domain 5 with a CD163-like homolog confers resistance of pigs to genotype 1 but not genotype 2 porcine reproductive and respiratory syndrome virus. J Virol.

[50] Salgado B, Rivas RB, Pinto D, Sonstegard TS, Carlson DF (2024). Genetically modified pigs lacking CD163 PSTII-domain-coding exon 13 are completely resistant to PRRSV infection. Antiviral Res.

[51] Zhang M, Han X, Osterrieder K, Veit M (2021). Palmitoylation of the envelope membrane proteins GP5 and M of porcine reproductive and respiratory syndrome virus is essential for virus growth. PLoS Pathog.

[52] Costers S, Lefebvre DJ, Delputte PL, Nauwynck HJ (2008). Porcine reproductive and respiratory syndrome virus modulates apoptosis during replication in alveolar macrophages. Arch Virol.

[53] Vocadlo DJ, Lowary TL, Bertozzi CR, Schnaar RL, Esko JD (2022). Essentials of Glycobiology.

[54] Elbein AD, Tropea JE, Mitchell M, Kaushal GP (1990). Kifunensine, a potent inhibitor of the glycoprotein processing mannosidase I. J Biol Chem.

[55] Yang Q, Hughes TA, Kelkar A, Yu X, Cheng K (2020). Inhibition of SARS-CoV-2 viral entry upon blocking N- and O-glycan elaboration. Elife.

[56] Li J, Tao S, Orlando R, Murtaugh MP (2015). N-glycosylation profiling of porcine reproductive and respiratory syndrome virus envelope glycoprotein 5. Virology.

[57] Kim HS, Kwang J, Yoon IJ, Joo HS, Frey ML (1993). Enhanced replication of porcine reproductive and respiratory syndrome (PRRS) virus in a homogeneous subpopulation of MA-104 cell line. Arch Virol.

[58] Yim-Im W, Huang H, Park J, Wang C, Calzada G (2021). Comparison of ZMAC and MARC-145 cell lines for improving porcine reproductive and respiratory syndrome virus isolation from clinical samples. J Clin Microbiol.

[59] Murtaugh MP, Stadejek T, Abrahante JE, Lam TTY, Leung FC-C (2010). The ever-expanding diversity of porcine reproductive and respiratory syndrome virus. Virus Res.

[60] Shi M, Lam TT-Y, Hon C-C, Hui RK-H, Faaberg KS (2010). Molecular epidemiology of PRRSV: a phylogenetic perspective. Virus Res.

[61] Yang H, Zhang J, Zhang X, Shi J, Pan Y (2018). CD163 knockout pigs are fully resistant to highly pathogenic porcine reproductive and respiratory syndrome virus. Antivir Res.

[62] Whitworth KM, Rowland RRR, Ewen CL, Trible BR, Kerrigan MA (2016). Gene-edited pigs are protected from porcine reproductive and respiratory syndrome virus. Nat Biotechnol.

[63] Xu K, Zhou Y, Mu Y, Liu Z, Hou S (2020). *CD163* and *pAPN* double-knockout pigs are resistant to PRRSV and TGEV and exhibit decreased susceptibility to PDCoV while maintaining normal production performance. Elife.

[64] Prather RS, Wells KD, Whitworth KM, Kerrigan MA, Samuel MS (2017). Knockout of maternal CD163 protects fetuses from infection with porcine reproductive and respiratory syndrome virus (PRRSV). Sci Rep.

[65] Esko JD, Bertozzi C, Schnaar RL (2017). Chemical Tools for Inhibiting Glycosylation.

[66] Xie J, Christiaens I, Yang B, Trus I, Devriendt B (2018). Preferential use of Siglec-1 or Siglec-10 by type 1 and type 2 PRRSV strains to infect PK15S1–CD163 and PK15S10–CD163 cells. Vet Res.

[67] Chang VT, Crispin M, Aricescu AR, Harvey DJ, Nettleship JE (2007). Glycoprotein structural genomics: solving the glycosylation problem. Structure.

[68] Allen JD, Watanabe Y, Chawla H, Newby ML, Crispin M (2021). Subtle influence of ACE2 glycan processing on SARS-CoV-2 recognition. J Mol Biol.

[69] Hanson SR, Culyba EK, Hsu T-L, Wong C-H, Kelly JW (2009). The core trisaccharide of an N-linked glycoprotein intrinsically accelerates folding and enhances stability. Proc Natl Acad Sci USA.

[70] Tseng Y-C, Wu C-Y, Liu M-L, Chen T-H, Chiang W-L (2019). Egg-based influenza split virus vaccine with monoglycosylation induces cross-strain protection against influenza virus infections. Proc Natl Acad Sci USA.

[71] Thaa B, Kaufer S, Neumann SA, Peibst B, Nauwynck H (2017). The complex co-translational processing of glycoprotein GP5 of type 1 porcine reproductive and respiratory syndrome virus. Virus Res.

[72] Wei Z, Lin T, Sun L, Li Y, Wang X (2012). N-linked glycosylation of GP5 of porcine reproductive and respiratory syndrome virus is critically important for virus replication *In Vivo*. J Virol.

[73] Bieberich E (2014). Glycobiology of the Nervous System.

[74] Lee HS, Qi Y, Im W (2015). Effects of N-glycosylation on protein conformation and dynamics: Protein Data Bank analysis and molecular dynamics simulation study. Sci Rep.

[75] Jayaprakash NG, Surolia A (2017). Role of glycosylation in nucleating protein folding and stability. Biochem J.

[76] Li Y, Liu D, Wang Y, Su W, Liu G (2021). The importance of glycans of viral and host proteins in enveloped virus infection. Front Immunol.

[77] Mathys L, Balzarini J (2015). Several N-glycans on the HIV envelope glycoprotein gp120 preferentially locate near disulphide bridges and are required for efficient infectivity and virus transmission. PLoS One.

[78] Tate MD, Brooks AG, Reading PC (2011). Specific sites of N-linked glycosylation on the hemagglutinin of H1N1 subtype influenza A virus determine sensitivity to inhibitors of the innate immune system and virulence in mice. J Immunol.

[79] Tate MD, Job ER, Brooks AG, Reading PC (2011). Glycosylation of the hemagglutinin modulates the sensitivity of H3N2 influenza viruses to innate proteins in airway secretions and virulence in mice. Virology.

[80] Bouvier NM, Palese P (2008). The biology of influenza viruses. Vaccine.

[81] Li Q, Wu J, Nie J, Zhang L, Hao H (2020). The impact of mutations in SARS-CoV-2 spike on viral infectivity and antigenicity. Cell.

[82] Azad T, Singaravelu R, Taha Z, Jamieson TR, Boulton S (2021). Nanoluciferase complementation-based bioreporter reveals the importance of N-linked glycosylation of SARS-CoV-2 S for viral entry. Mol Ther.

[83] Nugent MA (2022). The future of the COVID-19 pandemic: how good (or Bad) can the SARS-CoV2 spike protein get?. Cells.

[84] Knight RL, Schultz KLW, Kent RJ, Venkatesan M, Griffin DE (2009). Role of N-linked glycosylation for sindbis virus infection and replication in vertebrate and invertebrate systems. J Virol.

[85] Park D, Xu G, Barboza M, Shah IM, Wong M (2017). Enterocyte glycosylation is responsive to changes in extracellular conditions: implications for membrane functions. Glycobiology.

[86] Choi H-Y, Park H, Hong JK, Kim S-D, Kwon J-Y (2018). N-glycan remodeling using mannosidase inhibitors to increase high-mannose glycans on acid α-glucosidase in transgenic rice cell cultures. Sci Rep.

[87] Feng T, Zhang J, Chen Z, Pan W, Chen Z (2022). Glycosylation of viral proteins: implication in virus-host interaction and virulence. Virulence.

[88] Li W, Hulswit RJG, Widjaja I, Raj VS, McBride R (2017). Identification of sialic acid-binding function for the Middle East respiratory syndrome coronavirus spike glycoprotein. Proc Natl Acad Sci USA.

[89] Löfling J, Lyi SM, Parrish CR, Varki A (2013). Canine and feline parvoviruses preferentially recognize the non-human cell surface sialic acid N-glycolylneuraminic acid. Virology.

[90] Stencel-Baerenwald JE, Reiss K, Reiter DM, Stehle T, Dermody TS (2014). The sweet spot: defining virus-sialic acid interactions. Nat Rev Microbiol.

[91] Watanabe Y, Bowden TA, Wilson IA, Crispin M (2019). Exploitation of glycosylation in enveloped virus pathobiology. Biochim Biophys Acta Gen Subj.

[92] Lee KH, Ahn JI, Yu DH, Jeong HS, Lee SH (2001). Effect of N-glycosylation on ligand binding affinity of rat V1a vasopressin receptor. Biochem Biophys Res Commun.

[93] Zhong X, Kriz R, Seehra J, Kumar R (2004). N-linked glycosylation of platelet P2Y12 ADP receptor is essential for signal transduction but not for ligand binding or cell surface expression. FEBS Lett.

[94] Chattopadhyay S, Bagchi P, Dutta D, Mukherjee A, Kobayashi N (2010). Computational identification of post-translational modification sites and functional families reveal possible moonlighting role of rotaviral proteins. Bioinformation.

