# Supplementary material for: Role of N-linked glycosylation in porcine reproductive and respiratory syndrome virus (PRRSV) infection
Source: J Gen Virol. 2024 May 22;105(5):001994. doi: 10.1099/jgv.0.001994 (PMC11165596; doi:10.1099/jgv.0.001994)
Supplement: Uncited Supplementary Material 1. [file jgv-105-01994-s001.pdf]

Supplementary Information

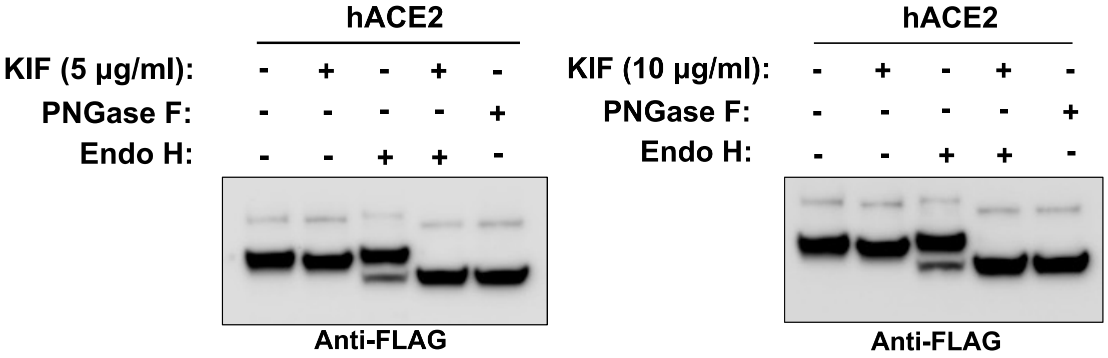

Figure S1. Western blots of hACE2 expression levels after kifunensine (KIF) treatment and Endo H digestion. Marc-145 cells were transfected with a plasmid expressing hACE2 (human ACE2) and treated with KIF at 5-10 µg/ml for 16 h before harvesting. Untreated or KIF-treated cell lysates were digested with Endo H or PNGase F and immunoblotted with anti-FLAG antibody.

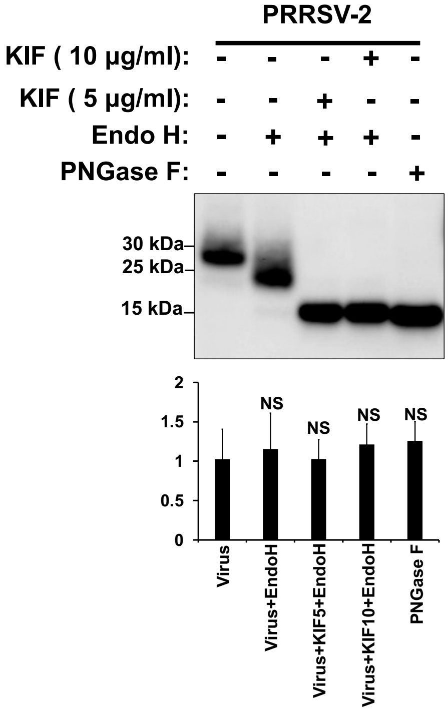

Figure S2. Western blot of GP5 expression levels of P129-GFP propagated in MARC-145 cells with KIF at 5-10 µg/ml and digested with Endo H or PNGase F. Proteins bands were detected as described in Figure 1B. Densitometry of GP5 bands is shown below the immunoblot. Fold changes are shown relative to lane 1 (Virus) and data are mean values ± standard deviation (SD) of three independent experiments (NS, not significant).

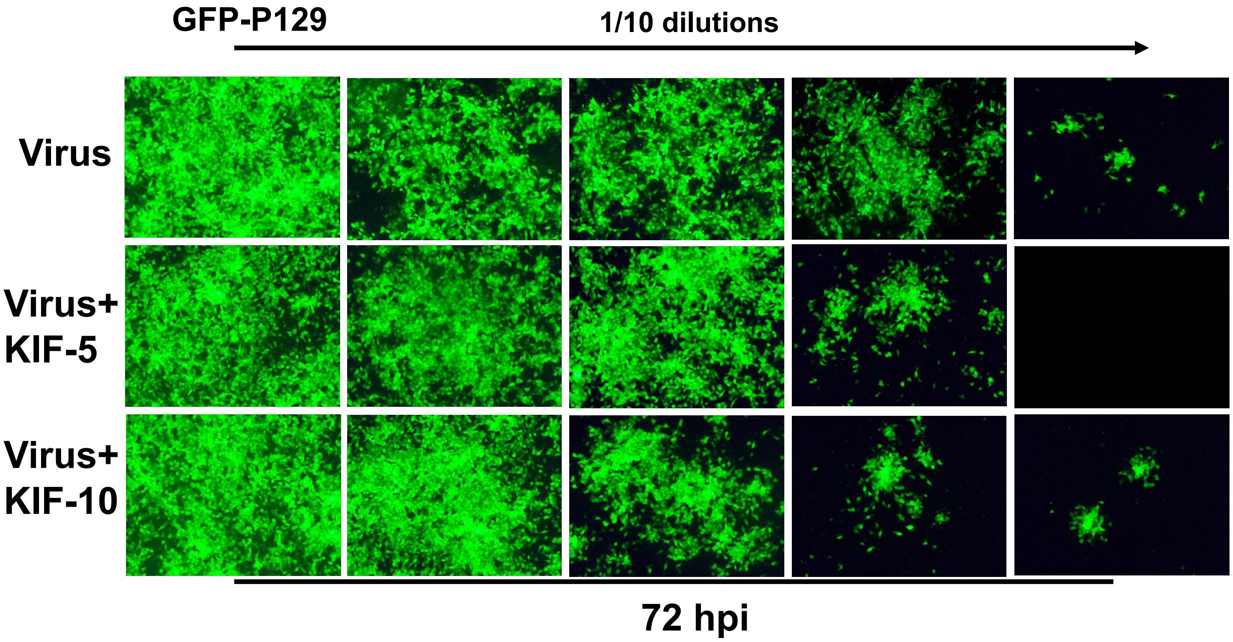

Figure S3. Effect of KIF on PRRSV-2 infection. MARC-145 cells were infected with different dilutions of P129-GFP virus propagated with KIF at 5-10 µg/ml. At 72 hours post-infection (hpi), the cells were fixed and visualized under a fluorescence microscope. Similar results were obtained in three separate experiments and representative data is shown.

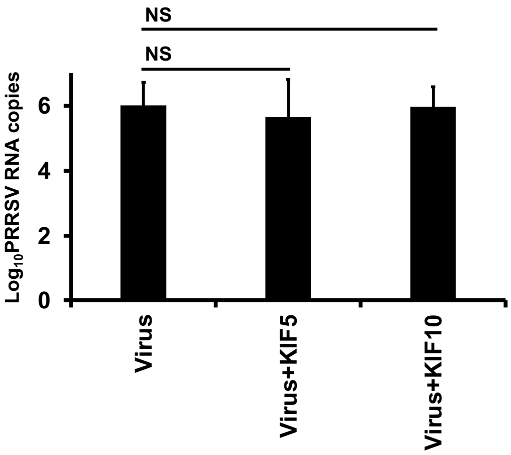

Figure S4. Effect of kifunensine (KIF) on PRRSV particle formation. PRRSV-2 (P129 isolate) was cultured in MARC-145 cells with or without KIF as indicated for 72h. The amount of progeny virus in culture supernatant was measured by quantitative real-time PCR as described in Methods. Results are shown as $\log_{10}$ copies per reaction. Results are means ± standard deviation (SD) values from three independent experiments (NS, not significant).

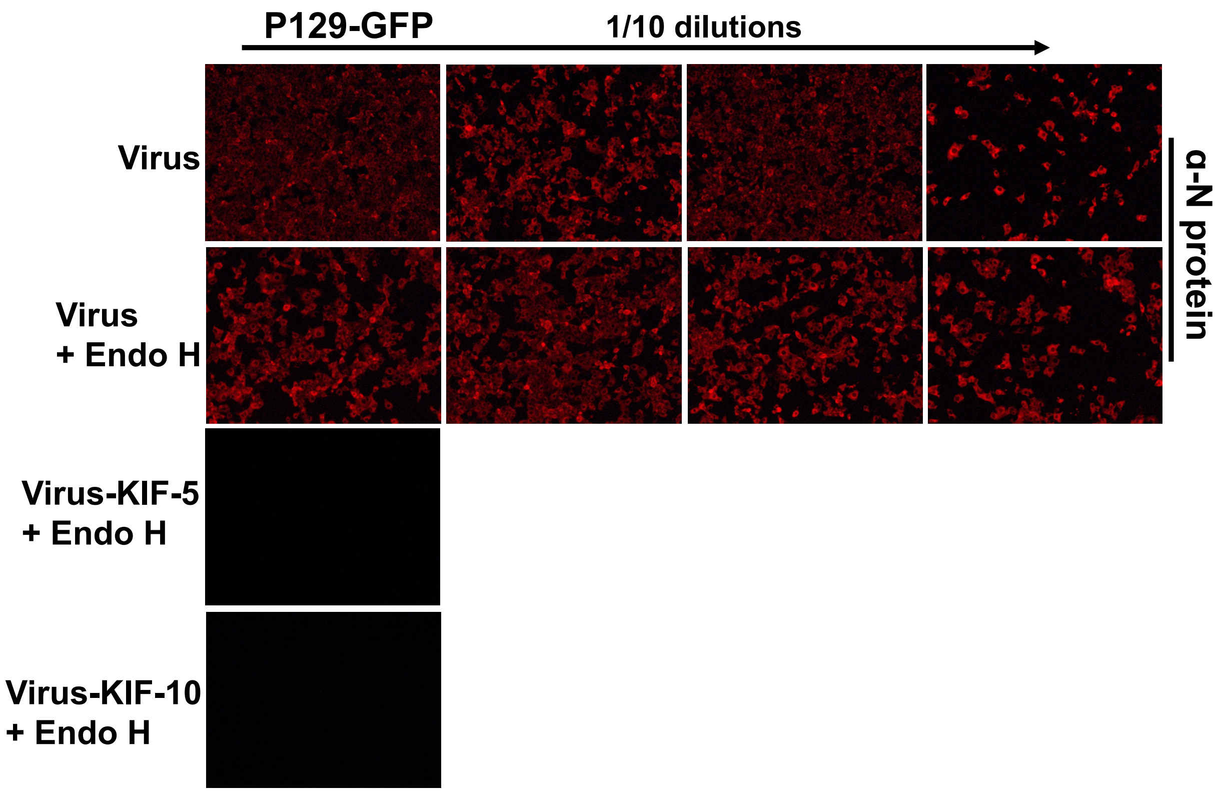

Figure S5. Effect of Endo H digestion on PRRSV infection. P129-GFP propagated in absence or presence of KIF at 5-10 μg/ml for 3 days was treated with Endo H at 37 °C for 1 h. Different dilutions of virus–enzyme mixtures (starting MOI=1) were inoculated onto MARC-145 cells at 37 °C for 1 h. After infection, cells were washed to remove enzyme and unbound virus, and further incubated for 72 h. Virus without enzyme treatment was incubated at 37 °C for 1 h to serve as a temperature stability control. To control for an enzyme effect on cells, Endo H incubated alone at 37 °C for 1 h was mixed with the temperature-stability control virus and the mixture was added to the cells. Cells were then fixed and stained with PRRSV N protein antibody, followed by Alexa 594-goat anti-mouse IgG (red). Similar results were obtained in three separate experiments and representative data is shown.

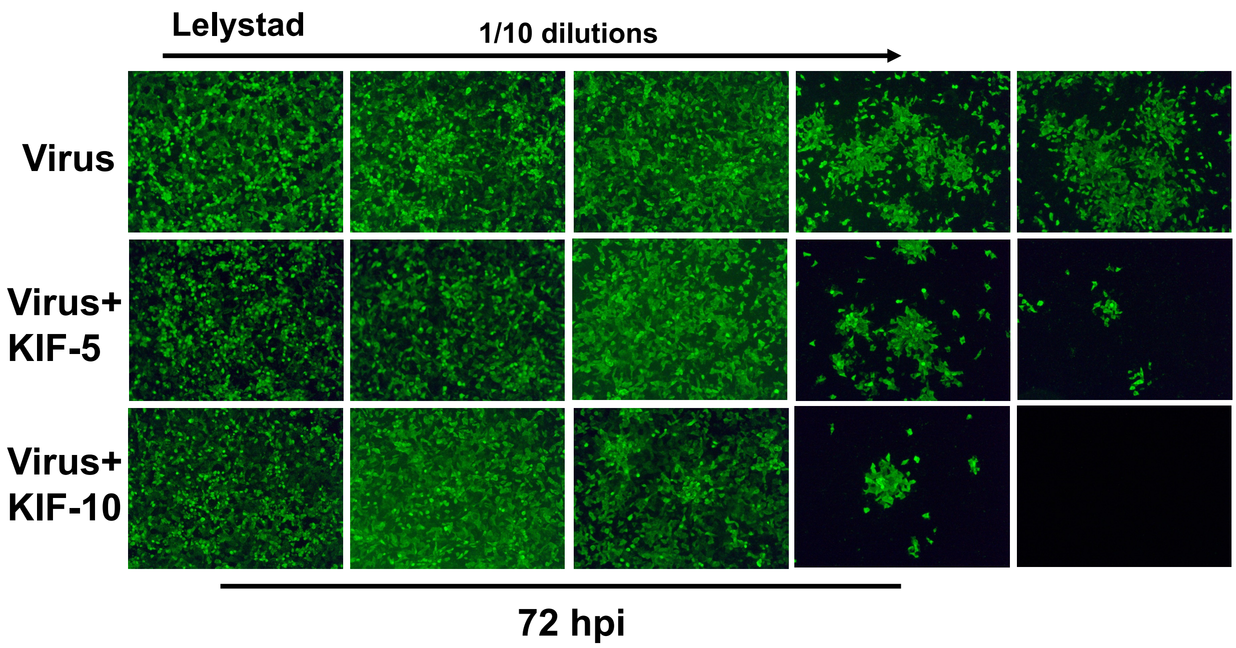

Figure S6. Effect of KIF on PRRSV-1 infection. MARC-145 cells were infected with different dilutions of Lelystad virus propagated with KIF at 5-10 µg/ml. At 72 hpi, the cells were fixed and stained with PRRSV N protein antibody, followed by Alexa 488-goat anti-mouse IgG (green). Similar results were obtained in three separate experiments and representative data is shown.

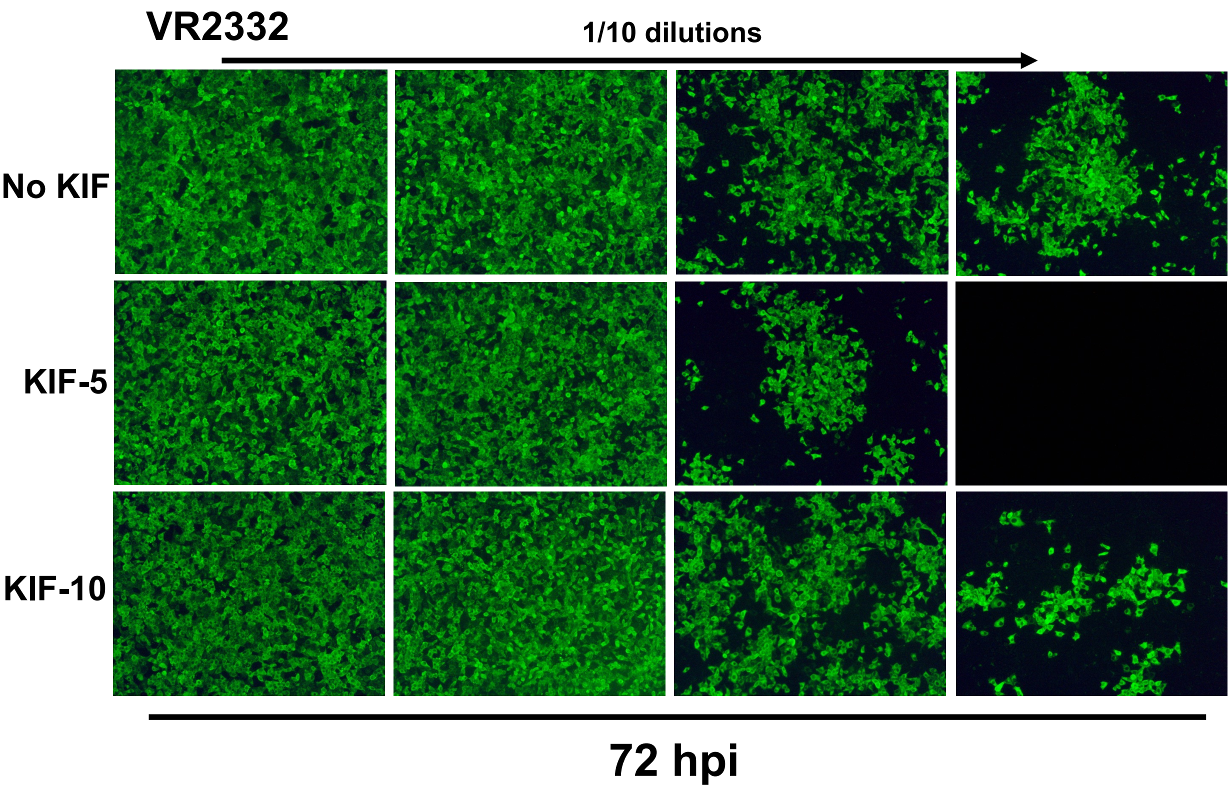

Figure S7. Effect of KIF on PRRSV-2 infection (VR2332). (A) MARC-145 cells were infected with different dilutions of VR2332 virus (starting MOI=1) in absence or presence of KIF at 5-10 µg/ml. At 72 hpi, the cells were fixed and stained as indicated in Figure S6. Similar results were obtained in three independent experiments and representative data is shown.

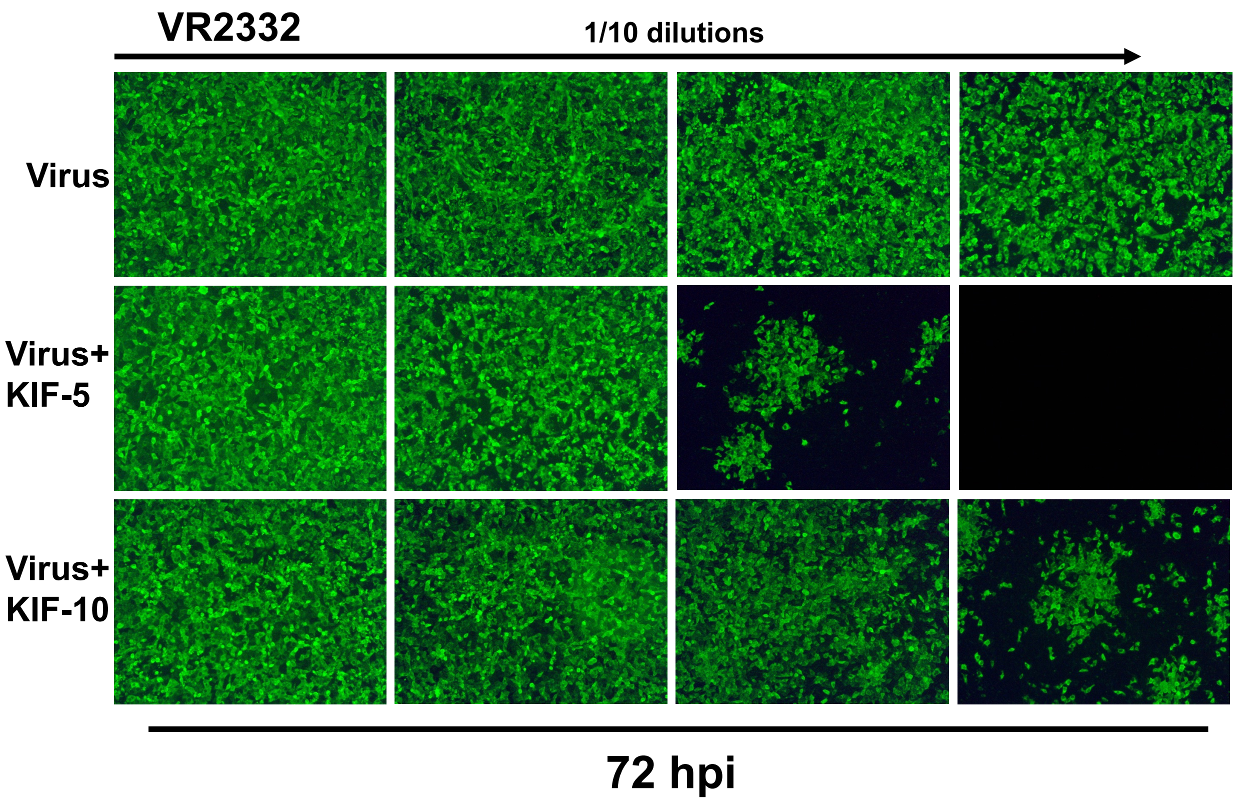

Figure S8. Effect of KIF on PRRSV-2 infection (VR2332). MARC-145 cells were infected with different dilutions of VR2332 virus propagated with KIF at 5-10 µg/ml. At 72 hpi, the cells were fixed and stained as indicated in Figure S6. Similar results were obtained in three separate experiments and representative data is shown.

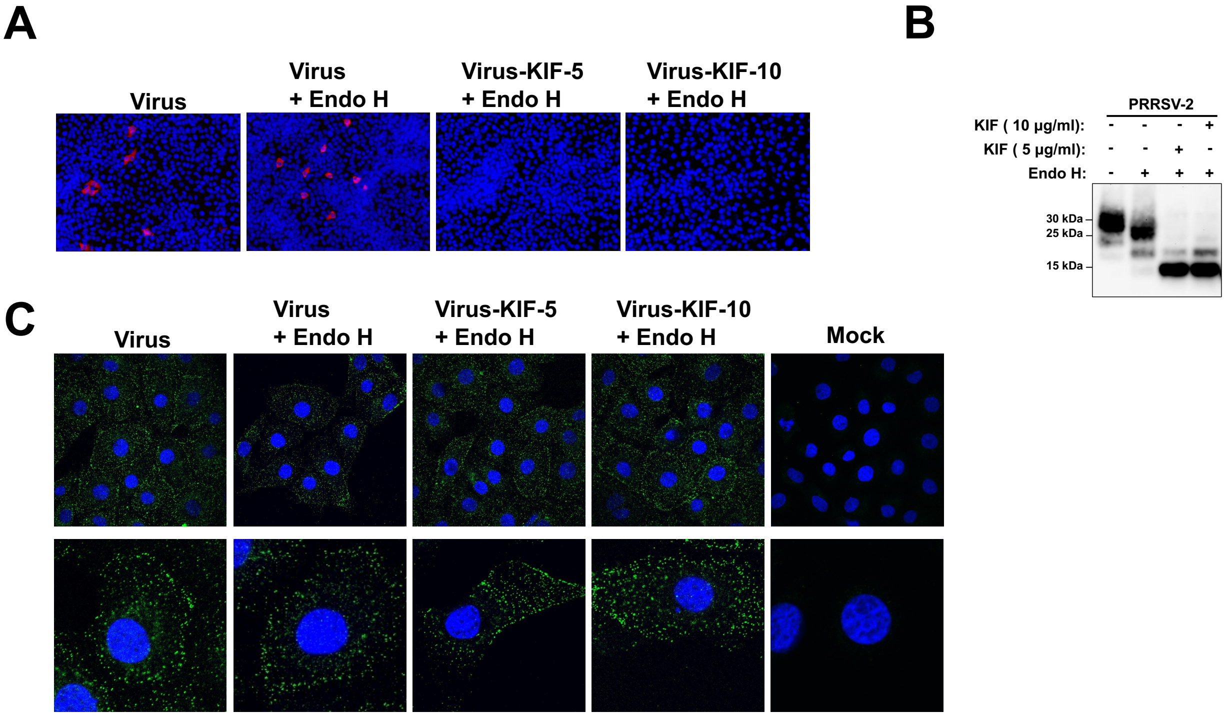

Figure S9. Effect of removal of N-glycans from PRRSV-2 (VR-2332) on viral entry and binding to the cell surface. (A) Effect of Endo H digestion on viral entry. PRRSV-2 propagated in absence or presence of KIF at 5-10 µg/ml for 3 days was treated with Endo H at 37 °C for 1 h. Different virus–enzyme mixtures (MOI=1) were inoculated onto MARC-145 cells at 37 °C for 1 h. Cells were then washed to remove enzyme and unbound virus, and further incubated for 9 h. Virus without enzyme treatment was incubated at 37 °C for 1 h to serve as a temperature stability control. To control for an enzyme effect on cells, Endo H incubated alone at 37 °C for 1 h was mixed with the temperature-stability control virus and the mixture was added to the cells. Cells were then fixed and stained with PRRSV N protein antibody, followed by Alexa 594-goat anti-mouse IgG (red). Nuclei were counterstained with DAPI (blue). Similar results were obtained in three separate experiments and representative data is shown. (B) Western blot of GP5 expression levels of VR2332 propagated in MARC-145 cells with KIF at 5-10 µg/ml and digested with Endo H. Proteins bands were detected with an anti-GP5 antibody. Similar results were obtained in three independent experiments and representative data is shown. (C) VR2332 was propagated in absence or presence of KIF and treated with EndoH as indicated above. Virus–enzyme mixtures (MOI=1) were inoculated onto MARC-145 cells at 4 °C for 1 h and subsequently washed to remove unbound virus. Virus without enzyme treatment was incubated at 37 °C for 1 h to serve as a temperature stability control. To control for an enzyme effect on cells, Endo H incubated alone at 37 °C for 1 h was mixed with the temperature-stability control virus and the mixture was added to the cells. Cells were then fixed, and stained with PRRSV N protein antibody, followed by Alexa 488-goat anti-mouse IgG (green). Nuclei were counterstained with DAPI (blue). Representative zoom images are shown on the bottom. Similar results were obtained in three separate experiments and representative data are shown.

Supplementary Table 1. Primers for the plasmids construction

Construct	Primer sequence
**CD163 N105Q**	F-TGGATGGGCTCAATTTAGTGCAG
	R-GTGGCTTTGATAGCAGTTG
**CD163 N139Q**	F-GGGAAAGCATCAGTGTACTCACC
	R-CATCCATCATGTTTGCAG
**CD163 N319Q**	F-TGGTCGAGTTCAAGCCAGTGAGG
	R-ATGGCAGTGACAGCAGTT
**CD163 N693Q**	F-ATCCCCGTGCCAATCATCATCCTC
	R-AGTGTCTGACTCTGGTTC
**CD163 N766Q**	F-ATGGGCCATTCAAGCCACTGGTTC
	R-CCACAGCTCAGCTGTTTG
**CD163 N936Q**	F-AGGAAACACTCAATGTTCTGGACG
	R-TCTTGAAGTCTTATTTTGTTG
**CD163 N1106Q**	F-GGATATGCTACAACCCTCAGGAG
	R-GTTTCATCTGCTTTCAGG

Supplementary Table 2. Primers for the plasmids construction

Construct	Primer sequence
**GP2 N178Q**	F-GACAGGTTCACAGGTAACCATAGTGTATAATAG
	R-ATGCGCAGGTTGTGTAGC
**GP2 N184Q**	F-CATAGTGTATCAGAGCACTTTGAATC
	R-GTTACATTTGAACCTGTC
**GP3 N29Q**	F-TGCGGGTTCCCAGACTACGTACT
	R-ACCACAGCACAACAAAAAG
**GP3 N42Q**	F-GGTTAGGGGCCAGTTTTCTTTCG
	R-AGCGGAAACCAAAAACAG
**GP3 N50Q**	F-ACTCACAGTGCAGTACACGGTGTG
	R-TCGAAAGAAAAATTGCCCC
**GP3 N131Q**	F- CGGGATAGGGCAGGTGAGTCGAG
	R- AATATCTCGGGGTGGAAC
**GP3 N152Q**	F-TGACGGGCAGCAGACCACCTTGC
	R-TGTTCGGCGCAGATGAGTTG
**GP3 N160Q**	F-TCGTCATGACCAGATTTCAGCCGTGTTTC
	R-GGCAAGGTGGTCTGCTGC
**GP3 N195Q**	F-GTTGGTTTTACAGGTCTCTTGGTTTC
	R-CACGAAGAAAAGAAGGGAC
**GP4 N37Q**	F-TATTAAGACCCAGACCACCGCAG
	R-TCCGCAAGACTCGAACTG
**GP4 N84Q**	F-CATCACAGCCCAGGTGACAGATG
	R-GTAACATACACGGGTGTC
**GP4 N120Q**	F-GGTATTTGGCCAGGTGTCAGGCATC
	R-ACCTTAAATCCCTTTTCACTC
**GP4 N130Q**	F-TGTGTGTGTCCAGTTTACCAGCTATGTC
	R-GCCACGATGCCTGACACA
**GP5 N30Q**	F-TGTGCTCGCCCAGGCCAGCAACG
	R-GCAAAACAGAACGGCACG
**GP5 N33Q**	F-CCAGGCCAGCCAGGACAGCAGCT
	R-GCGAGCACAGCAAAACAG
**GP5 N44Q**	F-GCTGATTTACCAGTTGACGCTATGTG
	R-TGTACATGGGAGCTGCTG
**GP5 N51Q**	F-ATGTGAGCTGCAGGGCACAGATTG
	R-AGCGTCAACTGGTAAATC
